# CRISPR-Cas9 high-throughput screening to study drug resistance in *Leishmania infantum*

**DOI:** 10.1128/mbio.00477-24

**Published:** 2024-06-12

**Authors:** Marine Queffeulou, Philippe Leprohon, Christopher Fernandez-Prada, Marc Ouellette, Ana María Mejía-Jaramillo

**Affiliations:** 1 Centre de Recherche en Infectiologie du Centre de Recherche du CHU Québec, Université Laval, Québec, Canada; 2 Département de Microbiologie, Infectiologie et Immunologie, Faculté de Médecine, Université Laval, Québec, Canada; National Institute of Allergy and Infectious Diseases Division of Intramural Research, Bethesda, Maryland, USA

**Keywords:** CRISPR-Cas9, drug resistance, *Leishmania*, next-generation sequencing, miltefosine, amphotericin B

## Abstract

Parasites of the genus *Leishmania* pose a global health threat with limited treatment options. New drugs are urgently needed, and genomic screens have the potential to accelerate target discovery, mode of action, and resistance mechanisms against these new drugs. We describe here our effort in developing a genome-wide CRISPR-Cas9 screen in *Leishmania*, an organism lacking a functional nonhomologous end joining system that must rely on microhomology-mediated end joining, single-strand annealing, or homologous recombination for repairing Cas9-induced double-stranded DNA breaks. A new vector for cloning and expressing single guide RNAs (sgRNAs) was designed and proven to be effective in a small pilot project while enriching specific sgRNAs during drug selection. We then developed a whole-genome library of 49,754 sgRNAs, targeting all the genes of *Leishmania infantum*. This library was transfected in *L. infantum* expressing Cas9, and these cells were selected for resistance to two antileishmanials, miltefosine and amphotericin B. The sgRNAs the most enriched in the miltefosine screen targeted the miltefosine transporter gene, but sgRNAs targeting genes coding for a RING-variant protein and a transmembrane protein were also enriched. The sgRNAs the most enriched by amphotericin B targeted the sterol 24 C methyltransferase genes and a hypothetical gene. Through gene disruption experiments, we proved that loss of function of these genes was associated with resistance. This study describes the feasibility of carrying out whole-genome CRISPR-Cas9 screens in *Leishmania* provided that a strong selective pressure is applied. Such a screen can be used for accelerating the development of urgently needed antileishmanial drugs.

**IMPORTANCE:**

Leishmaniasis, a global health threat, lacks adequate treatment options and drug resistance exacerbates the challenge. This study introduces a CRISPR-Cas9 screening approach in *Leishmania infantum*, unraveling mechanisms of drug resistance at a genome-wide scale. Our screen was applied against two main antileishmanial drugs, and guides were enriched upon drug selection. These guides targeted known and new targets, hence validating the use of this screen against *Leishmania*. This strategy provides a powerful tool to expedite drug discovery as well as potential therapeutic targets against this neglected tropical disease.

## INTRODUCTION

Leishmaniasis, caused by parasites of the genus *Leishmania,* is endemic in 98 countries. The available treatments for human leishmaniasis are limited, with only five imperfect licensed drugs (antimonials, miltefosine, paromomycin, amphotericin B, and pentamidine) associated with toxicity, high cost, or difficulty in administration. Additionally, the misuse of drugs paired with the extensive genome plasticity of *Leishmania* has led to drug resistance (1).

Antimicrobial drug resistance represents a worldwide threat, complicating the treatment of infectious diseases including against leishmaniasis. The development of new drugs is urgently needed, and the implementation of cutting-edge strategies may facilitate the identification of new bioactive molecules but also a holistic understanding of their mode of action, their target(s), and strategies used by the pathogens to resist drug action (2). The use of whole-genome sequencing has accelerated our understanding of resistance mechanisms in *Leishmania* (3). The combination of *in vitro* drug evolution with new drug candidates and genome sequencing has helped in highlighting new drug targets (4–7). New whole genomic screens based on overexpression or chemical mutagenesis were also developed to expedite the discovery of gene targets and resistance mechanisms in *Leishmania* (8–11).

The bacterial CRISPR (clustered regularly interspaced short palindromic repeat)-Cas9 system has been transformative where it can mediate efficient genome editing in a broad range of organisms. The CRISPR-Cas9 technology was adapted early into *Leishmania* research (12, 13). It has been used to induce a variety of modifications including single nucleotide polymorphisms, base insertion or deletion, gene knock-in, or gene deletion (14–18). In most organisms, double-stranded DNA breaks, as mediated by Cas9, are repaired either by homologous recombination (HR), microhomology-mediated end joining (MMEJ), single-strand annealing (SSA), or nonhomologous end joining (NHEJ). Whole-genome screens using CRISPR-Cas9 are facilitated in organisms with NHEJ and for example this has allowed CRISPR-Cas9 genome-wide screening in *Toxoplasma gondii* (16). However, only a few NHEJ factors are present in *Leishmania* (MRE11, Ku70/Ku80, and APTX) while Artemis, XRCC4, and the DNA ligase IV are absent (17). In contrast, MMEJ is fully functional in *Leishmania* (18, 19), and while genome-wide CRISPR-Cas9 loss-of-function screens may not be as effective as in organisms proficient in NHEJ, it should be theoretically possible in *Leishmania*.

We, thus, present here our efforts in developing a high-throughput strategy using CRISPR-Cas9 in *L. infantum*. We built cloning vectors (named DD and DD-a) compatible with Illumina sequencing that were used for generating sgRNA libraries made of six sgRNAs per parasite coding sequence (CDS) that were independently cloned under the control of a ribosomal RNA (rRNA) promoter. These libraries were transfected in *L. infantum* expressing Cas9 and the populations were screened with antileishmanial drugs. This strategy allowed the identification of known drug targets but also of new genes, whose involvement in drug resistance was experimentally validated. This new screen for *Leishmania* adds to our arsenal of tools to expedite our understanding of drug resistance of urgently needed new drugs.

## RESULTS

### Suitability of a new vector toward CRISPR-Cas9 screens

**Fig 1:**
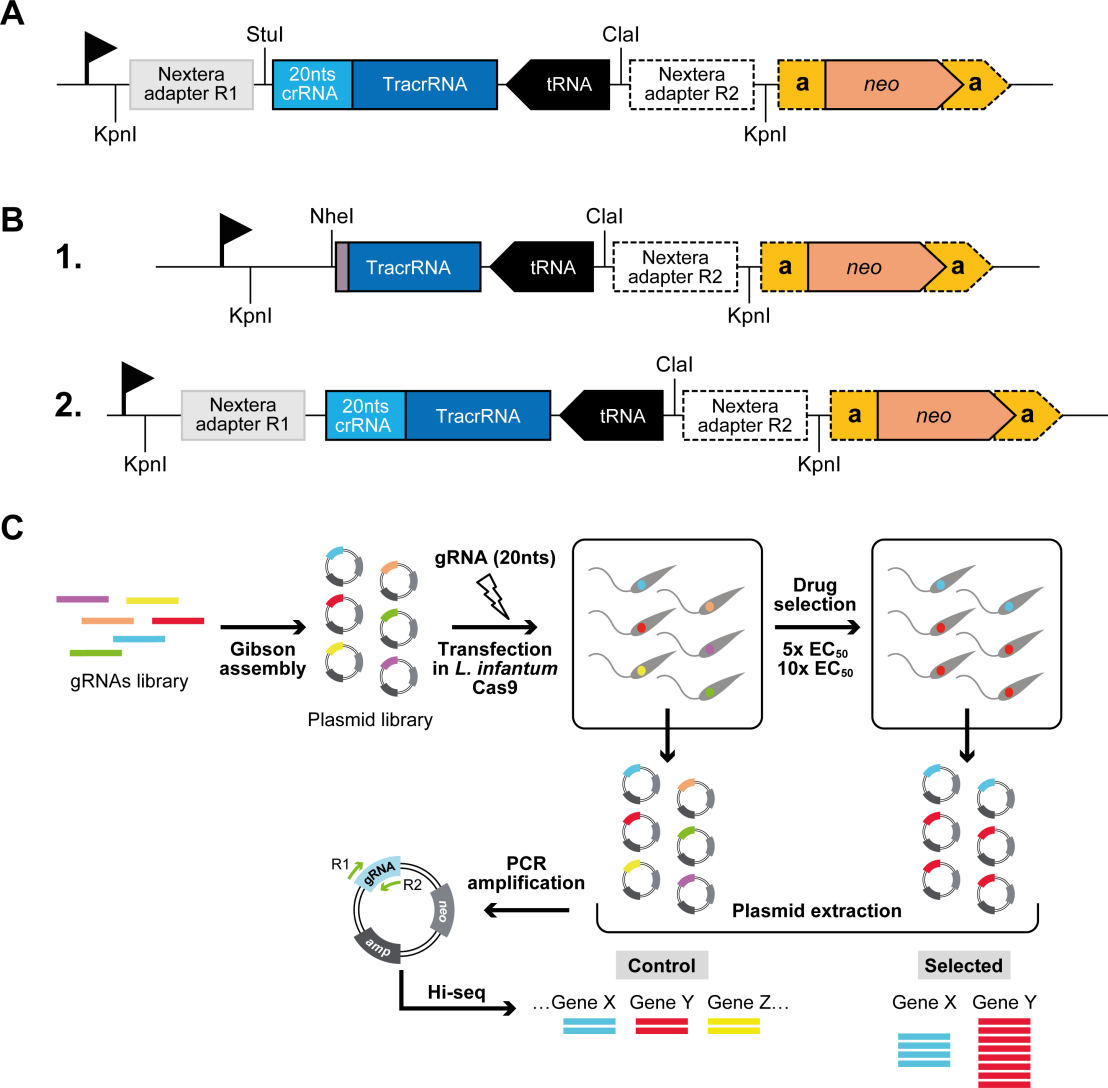
Strategy used to implement the CRISPR-Cas9 libraries in *Leishmania infantum*. (**A**) Representation of the DD vector used to express the sgRNAs in the mini library experiments and gene candidate validation tests. Are shown (not to scale) the *neomycin phosphotransferase* (*neo*) marker with the *alpha-tubulin* intergenic regions (α); the ribosomal promoter (flag); an antisense tRNA used as a terminator; two Illumina Nextera adapters R1 and R2 flanked by *Kpn*I restriction sites, a crRNA sequence (blue) and a TracrRNA (dark blue). (**B**) Representation of the DD-a vector used to express the sgRNAs for the whole genome screen before (1) and after (2) cloning the 20 nts crRNA and Nextera adapter R1 by Gibson assembly. Here, the TracrRNA is truncated in (1), missing the sequence GTTTTAGAG at its 5′ end, but recover its full length in (2) after Gibson assembly cloning (see also Fig. S3). (**C**) Overview of the whole-genome CRISPR-Cas9 approach in *Leishmania infantum*.

**Fig 2:**
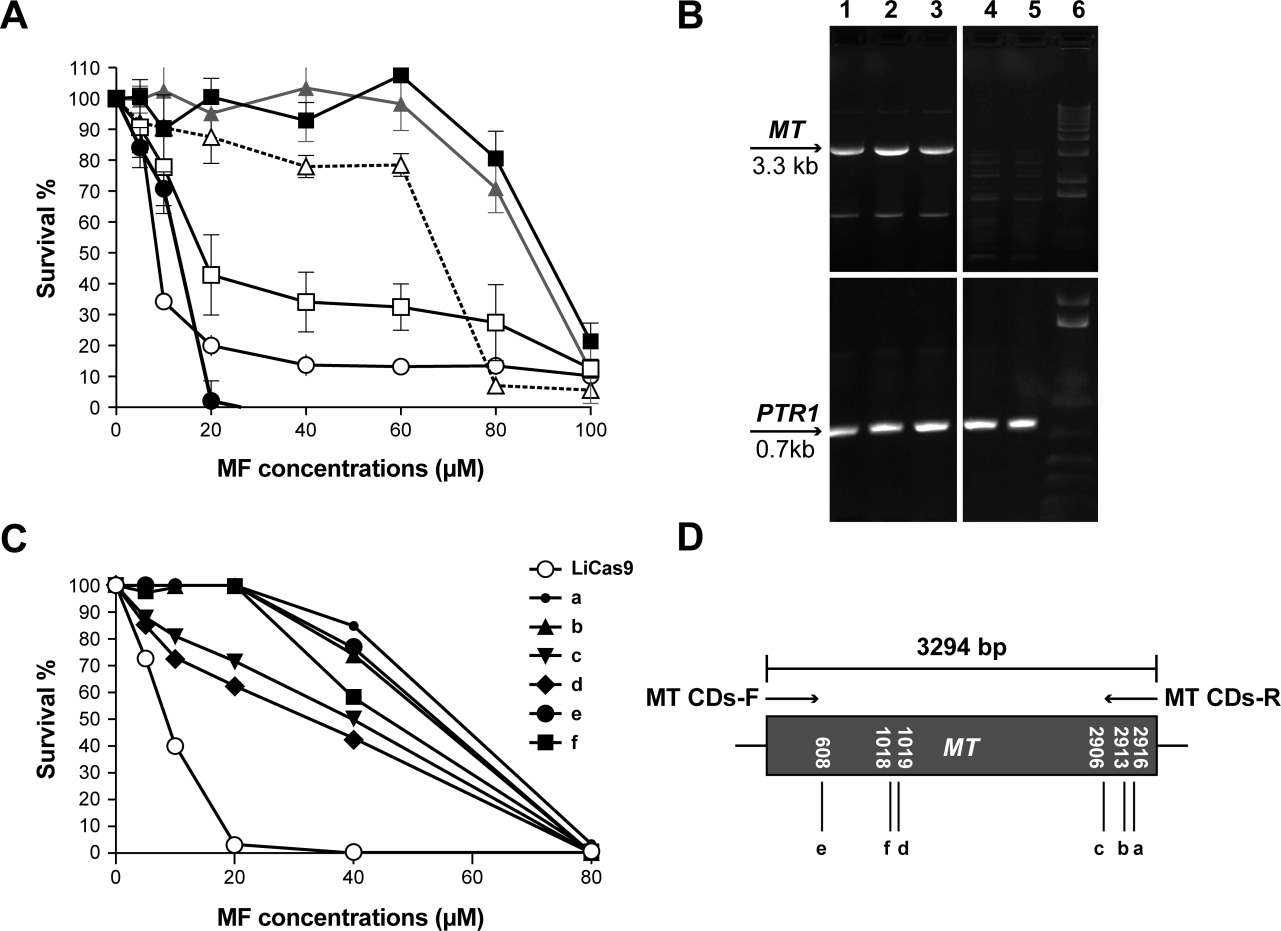
Targeting the miltefosine transporter gene *MT* using the DD vector for CRISPR-Cas9 mediated gene deletion. (A) Miltefosine susceptibility of Cas9 expressing *L. infantum* (○); of Cas9 expressing *L. infantum* transfected with empty DD selected with G418 and 10 × MF EC_50_ (●); of Cas9 expressing *L. infantum* transfected with DD-MT-1 either initially selected with G418 alone (□) or with G418 and 10 × MF EC50 (■); of Cas9 expressing *L. infantum* transfected with the MT-1 synthetic gRNA either with no drug selection (△) or selected with 10 × MF EC50 (▲). (B) PCR amplification of the *MT* gene open reading frame (upper gel) and of *PTR1* gene as control (lower gel). *L. infantum* (1); Cas9-expressing *L. infantum* (2); Cas9-expressing *L. infantum* transfected with DD-MT-1 and selected with G418 alone (3), with G418 and 5 × MF EC_50_ (4), or with G418 and 10 × MF EC50 (5); 1 kb plus DNA ladder (6). The gels have been spliced between lanes 3 and 4 to remove non-relevant lanes from the original gel. (C) Transfection of DD-a-MTs in Cas9-expressing *L. infantum* selected with 10 × MF EC_50_ prior to the measurement of MF EC_50_ of the transformants. Cas9-expressing *L. infantum* (LiCas9) and Cas9-expressing *L. infantum* transfected with DD-a-MT-1 (a), DD-a-MT-2 (b), DD-a-MT-3 (c), DD-a-MT-4 (d), DD-a-MT-5 (**e**), or DD-a-MT-6 (f). All growth curves were done once with four technical replicates, and the standard deviation (SD) was included. (D) Schematic representation of the *MT* locus with the position of the six sgRNAs indicated along the gene as well as the forward (F) and reverse (R) primers.

We used CRISPR-Cas9 in the past (10, 20, 21) usually by electroporating directly the crRNAs (along with a TracrRNA) and always using a DNA repair template. For whole-genome screens, there is no repair template, and a vector is needed for the expression of sgRNAs targeting the whole genome. Selection will lead to loss-of-function gene deletion mediated by MMEJ or SSA in target genes. As a first proof of principle, we tested the sgRNA MT-1 (Table S1) either directly as a sgRNA or cloned in the DD vector (Fig. 1). This sgRNA targets the *MT* gene which codes for the phospholipid-transporting ATPase 1-like protein (*LINF_130020800*) whose loss-of-function produces miltefosine (MF) resistance (22, 23). Cas9-expressing *L. infantum* were transfected with the DD-MT-1 vector. In the absence of MF selection, the transfectants were not resistant to MF (Fig. 2A). However, if MF was added after the transfection (at 10 × the EC_50_ of *L. infantum*), growing parasites were shown to be highly resistant to MF when tested afterward (Fig. 2A). In contrast, no growth was observed in Cas9-expressing parasites transfected with the empty DD vector and selected with 10 × MF EC_50_. It would appear that direct electroporation of the MT-1 sgRNA in Cas9-expressing cells was more effective, however, since in this case even cells grown without the MF selection were resistant to MF when assessed (Fig. 2A). Similar results were observed if cells were selected at 5 × the EC_50_ of MF instead of 10 × (Fig. S1A). The DD vector is, thus, functional provided that strong pressure is in play to select for parasites whose *MT* gene has been edited. Indeed, the *MT* locus was deleted in Cas9-expressing parasites transfected with DD-MT-1 when selected with 5 × or 10 × EC_50_ of MF as determined by PCR (Fig. 2B) or Southern blots (Fig. S1B). The extent of the deletion was not studied in detail, but it appears that different MMEJ or SSA events occurred as evidenced by PCR amplification where several low molecular weight bands were observed (Fig. S1C). While Cas9 vectors may lead to cellular toxicity (24), this was not the case with the DD vector (Fig. S2).

We also validated the DD-a vector, which is similar to DD but compatible with Gibson assembly cloning (Fig. 1B; Fig. S3). We designed six guides (MT-1 to MT-6, Table S1) targeting the *MT* gene that were independently cloned into the DD-a vector. Their individual transfection into Cas9-expressing *L. infantum* produced MF-resistant lines, provided that these were prior selected with 10 × the EC_50_ of MF (Fig. 2C). The *MT* locus was deleted in parasites transfected with the guides MT-1, MT-2, MT-5, and MT-6, while it was likely mutated in cells transfected with guides MT-3 and MT-4 (Fig. S4).

### Proof of concept for CRISPR-Cas9 using a small library of sgRNAs

**Fig 3:**
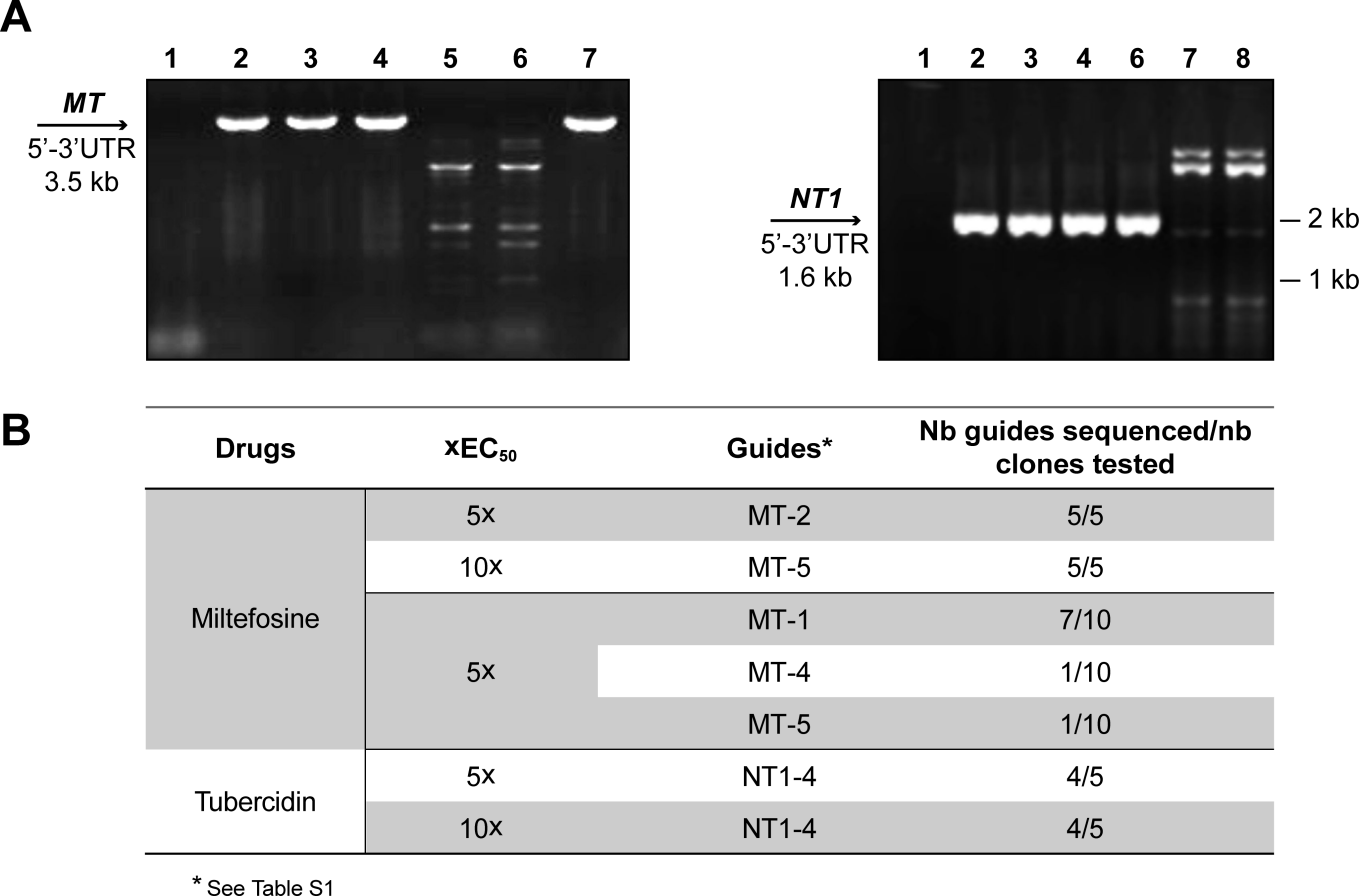
Small scale proof of principle for CRISPR-Cas9 dominant negative screen. A mini library of 24 sgRNAs cloned into the DD vector was transfected in Cas9 expressing *L. infantum* and selected with drugs. (**A**) The genomic DNAs of growing parasites were used as templates for the amplification of the *MT* (left) or *NT1* (right) loci using primers annealing to their untranslated regions (1), water; (2), Cas9-expressing *L. infantum*; (3) Cas9-expressing *L. infantum* transfected with the DD library; Cas9-expressing *L. infantum* transfected with the DD library selected with 10× SbIII EC_50_ (4), with 5 × MF EC_50_ (5), with 10 × MF EC_50_ (6), with 5 × tubercidin EC_50_ (7), or with 10× tubercidin EC_50_ (8). (**B**) The DD vectors of parasites growing in 5× or 10× the EC_50_ of the drugs were isolated, and the specific sgRNAs they encoded were identified by Sanger sequencing. The sgRNA sequences are listed in Table S1. The names and sequences of the sgRNAs recovered from clones tested prior to transfection (i.e., *E. coli*) and after transfection in *L. infantum* but without drug selection are available in Table S2.

For proof of concept, we used a pool of 24 sgRNAs targeting four genes, including the six *MT* sgRNAs mentioned above, as well as six sgRNAs targeting each of the Aquaglyceroporin 1 gene *AQP1* (*LINF_310005100*) (25), the multicopy nucleoside transporter 1 gene *NT1* (*LINF_150019900*, *LINF_150020100, LINF_150020000*, *LINF_150020200*) (26), or the thymidine kinase gene (*LINF_210020200*) (27) (Table S1). We amplified by PCR the 24 sgRNAs and integrated them into the DD vector independently, which we then pooled. After confirming the presence of various sgRNAs in this mini-library by Sanger sequencing, we transfected it in Cas9-expressing *L. infantum* (Table S2). The transfectants were selected first with 5 × or 10 × EC_50_ of MF. By PCR, the *MT* gene was intact in unselected control cells transfected with the mini-library (Fig. 3A, lane 3, left panel) or in cells selected with SbIII or tubercidin (Fig. 3A, lanes 4 and 7, left panel). In contrast, the *MT* locus was deleted in cells selected with MF at 5 × or 10 × EC_50_ (Fig. 3A, lanes 5 and 6, left panel). The parasites from the latter two populations were resistant to MF (Fig. S5A). We isolated their DD vectors (along with the Cas9 episome), amplified the sgRNAs by PCR, and cloned the PCR products into pGEM-T-easy and transformed into *E. coli*. Five clones derived from both the 5 × and 10 × MF EC_50_ selected cells were sequenced by the Sanger method. The five clones derived from cells selected with 5 × the MF EC_50_ contained the MT-2 sgRNA while the five clones selected at 10× harbored the MT-5 guide (Fig. 3B). Since all six MT guides were functional in producing resistant isolates (Fig. 2C), we were intrigued that, in both cases, a single sgRNA was enriched. We, thus, replicated this small-scale screen, but this time selecting only with 5 × the EC_50_ of MF. We isolated the DD vectors from the population growing with MF, cloned their sgRNAs as described above, and sent 10 clones for Sanger sequencing. Nine of the 10 clones contained a sgRNA directed at *MT,* but this time, 3 different *MT* sgRNAs were enriched (Fig. 3B). The sequence obtained from the last clone was pGEM-T-easy without gRNA insertion.

The four genes from our pilot work were not chosen at random but picked up because their loss-of-function was associated with resistance. Indeed, the nucleoside transporter *NT1* was found deleted in tubercidinresistant parasites (28). We, thus, tested whether sgRNAs that specifically targeted *NT1* could be enriched by tubercidin. The *NT1* gene was intact in control cells transfected with the mini-library but not selected with drugs (Fig. 3A, lane 3, right panel), or in cells selected with SbIII (Fig. 3A, lane 4, right panel) or MF (Fig. 3A, lane 6, right panel). However, it was deleted if cells were selected with tubercidin at both 5 × and 10× its EC_50_ (Fig. 3A, lanes 7 and 8, right panel). The parasite populations with an edited *NT1* were resistant to tubercidin (Fig. S5B). We isolated their DD vectors and the sgRNAs were characterized by Sanger sequencing. For both selection, four of the five clones tested contained the NT1-4 sgRNA (Fig. 3B). The remaining clone at 5 × and 10 × tubercidin EC_50_ harbored the AQP1-6 sgRNA. We have not tested all six *NT1* guides independently but the NT1-1 and the NT1-4 sgRNAs were found to be functional and to target appropriately *NT1* when independently transfected as single gRNAs, leading to resistance to tubercidin (Fig. S6).

### CRISPR-Cas9 whole-genome screen in *Leishmania*

Our pilot work indicated that we could pinpoint genes targeted by sgRNAs provided that a strong drug pressure is applied (Fig. 2 and 3). We, thus, constructed in the DD-a vector a library of 49,754 sgRNAs composed of six sgRNAs per coding sequence of *L. infantum* and of 500 non-targeting control sgRNAs (Fig. 1; Table S3), to assess for library specificity. This library was transfected in *L. infantum* using 25 nucleofections that were combined. Vectors were extracted from transfected cells and sgRNAs were amplified by PCR. More than 95% of the sgRNAs from the library were detected among *Leishmania* transfectants (Fig. S7A). The skew ratio between the top 10% most abundant sgRNAs to the 10% less abundant sgRNAs was around 3, indicating a narrow distribution of read counts between sgRNAs and library evenness among these baseline transfectants (Fig. S7B).

**Fig 4:**
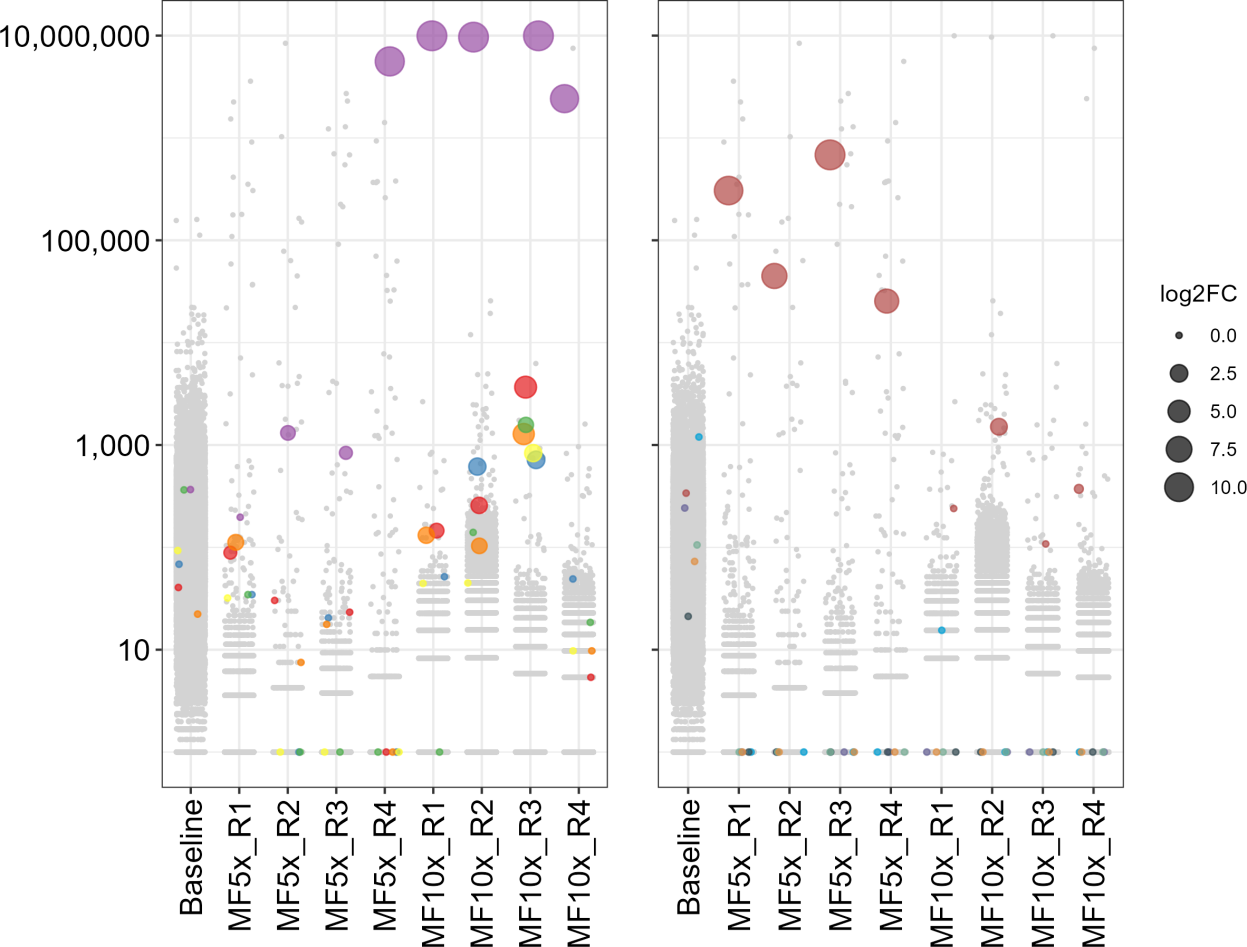
Abundance of sgRNAs in the CRISPR-Cas9 whole genome screen selected for miltefosine resistance. The abundance of sgRNAs identified by sequencing the DD-a vectors recovered from transfected parasites at baseline or after selection with 5 × and 10 × the EC_50_ of MF are shown. The screen was performed in four replicates (R1–R4). Each dot represents a sgRNA whose abundance in read count (normalized for library size to 10M total reads) is indicated by the *y*-axis. Colored dots indicate sgRNAs targeting the *MT* gene (left panel) or the RING-variant domain containing gene *LINF_310005200* (right panel), one color for each of the six different sgRNAs targeting the genes. The log2 fold change (log2FC) in abundance for these sgRNAs compared to the baseline is shown by dot size. Gray dots correspond to the bulk of sgRNAs.

**Fig 5:**
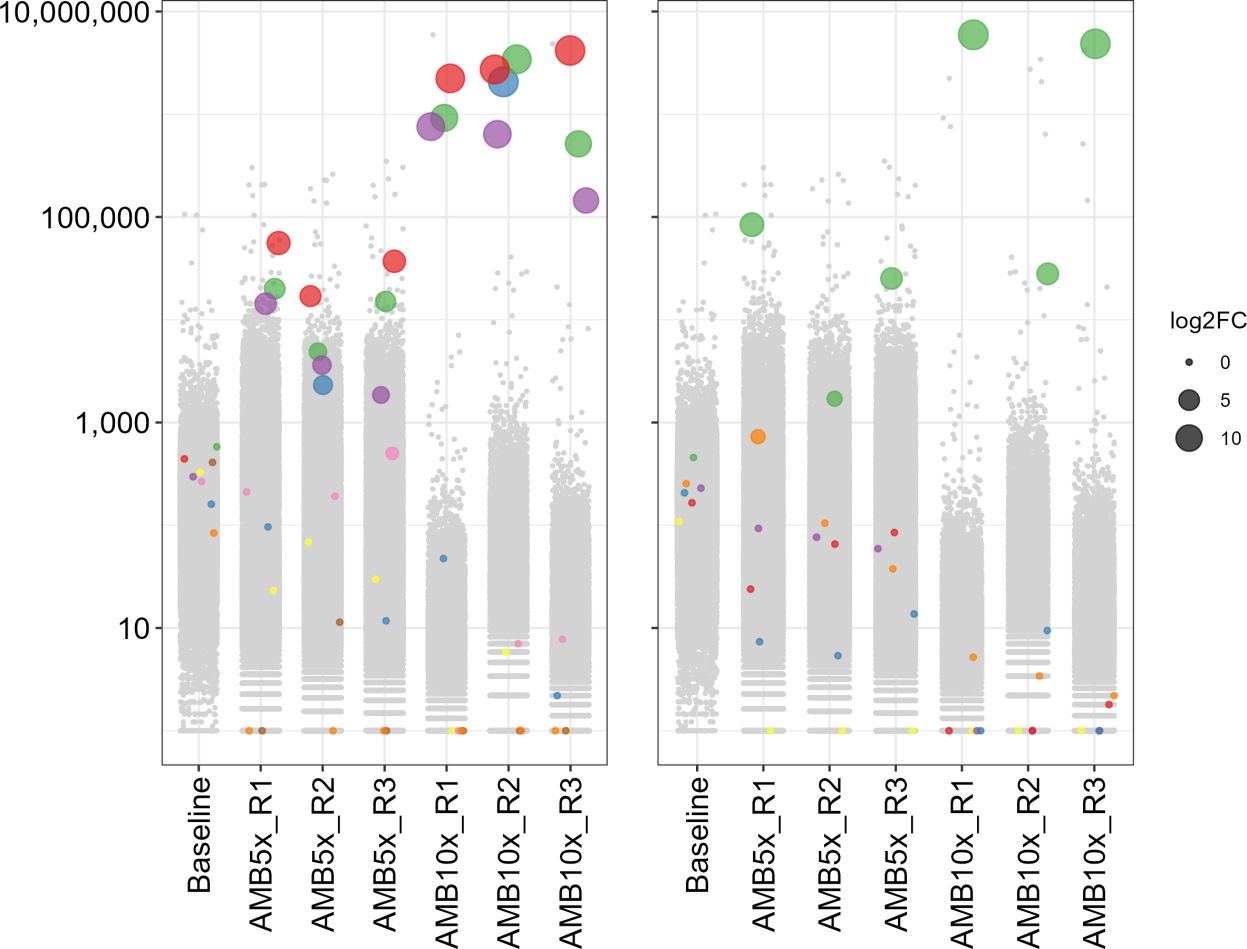
Abundance of sgRNAs in the CRISPR-Cas9 whole-genome screen selected for amphotericin B resistance. The abundance of sgRNAs identified by sequencing the DD-a vectors recovered from transfected parasites at baseline or after selection with 5 × and 10 × the EC_50_ of AMB are shown. The screen was performed in three biological replicates (R1–R3). Each dot represents a sgRNA whose abundance in read count (normalized for library size to 10M total reads) is indicated on the *y*-axis. Colored dots indicate sgRNAs targeting the *SMT* genes (left panel) or the hypothetical protein coding gene *LINF_350014000* (right panel), one color for each of the six different sgRNAs targeting the genes. The log2 fold change (log2FC) in abundance for these sgRNAs compared to the baseline is shown by dot size. Gray dots correspond to the bulk of sgRNAs.

We tested this whole-genome library against the antileishmanial drugs MF and amphotericin B (AMB). Transfectants were selected with 5 × or 10 × the EC_50_ of the drug, and once these grew at an OD_600_ of 0.5–0.6, their DD-a plasmids were isolated, and the sgRNAs were amplified from the extracted plasmids and sequenced. Sequencing reads were assigned to sgRNAs based on their sequence, yielding sgRNA counts which were fed to the MAGeCK software which reported normalized counts and whether sgRNAs were enriched (Table S4). In this study, we focused on genes whose sgRNA counts were enriched in all biological replicates of a given selection and at a significant level (Log2 fold change ≥ 8; FDR ≤ 0.05), to minimize studying artifacts. Although we showed that all the sgRNAs targeting *MT* were functional (Fig. 2C), only one sgRNA out of the six (LinJ.13.1590_619160-v2; Table S3) was enriched after MF selection, nearing a 1,000-fold enrichment compared to baseline in all four replicates selected at 10 × the MF EC_50_ and in replicate 4 (R4) of the 5 × MF selection (Fig. 4, left panel and Table S4). Besides *MT*, one sgRNA targeting the RING-variant domain protein gene *LINF_310005200* (LinJ.31.0040_14281-v2; Table S2) was found to have increased read counts in all four replicates selected with 5 × MF EC_50_, especially for replicates R1 and R3 which showed an >100-fold enrichment compared to the baseline (Fig. 4, right panel and Table S4). This sgRNA displayed a less noticeable enrichment in cells selected with 10 × MF EC_50_ however, its abundance remaining relatively stable compared to the baseline while most other sgRNAs decreased in abundance (Fig. 4, right panel). Lastly, a sgRNA targeting the gene LINF_200007400 (LinJ.20.0260_79976-v2) coding for a protein whose sole annotation is to have a transmembrane domain was significantly enriched in cells selected at 5 × MF EC_50_ (Fig. S8; Table S4). In the AMB screen, we observed in all three replicates selected with 10 × AMB EC_50_ an enrichment of more than 1,000-fold for 3 out of 6 sgRNAs (LinJ.36.2510/2520_496-man; LinJ.36.2510_725_revcom-man; LinJ.36.2510/2520_70_revcom-man; Table S3) targeting the sterol 24 C methyltransferase *SMT* genes *LINF_360031200* and *LINF_360031300* (Fig. 5, left panel and Table S4), which differ by a single nucleotide in their coding sequences. The same three sgRNAs were also enriched in the three replicates selected with 5 × AMB EC_50_, but to a lower level (Fig. 5, left panel). One sgRNA (LinJ.35.0950_401866-v2; Table S3) out of the six targeting the gene *LINF_350014000* coding for a hypothetical protein was 1,000-fold enriched in 2 of the 3 replicates selected with 10 × AMB EC_50_, and enriched by 10 to 20-fold in the other replicate and in the three replicates selected at 5 × AMB EC_50_ (Fig. 5, right panel). The latter concentration was probably not a strong enough selective pressure however, as some control gRNAs not targeting the *Leishmania* genome remained detectable while all were eliminated from the population selected at 10 × AMB EC_50_ (or with MF), which is expected for a drug resistance screen (Fig. S9).

### Validation of genes highlighted by the CRISPR-Cas9 screen in drug resistance

**Fig 6:**
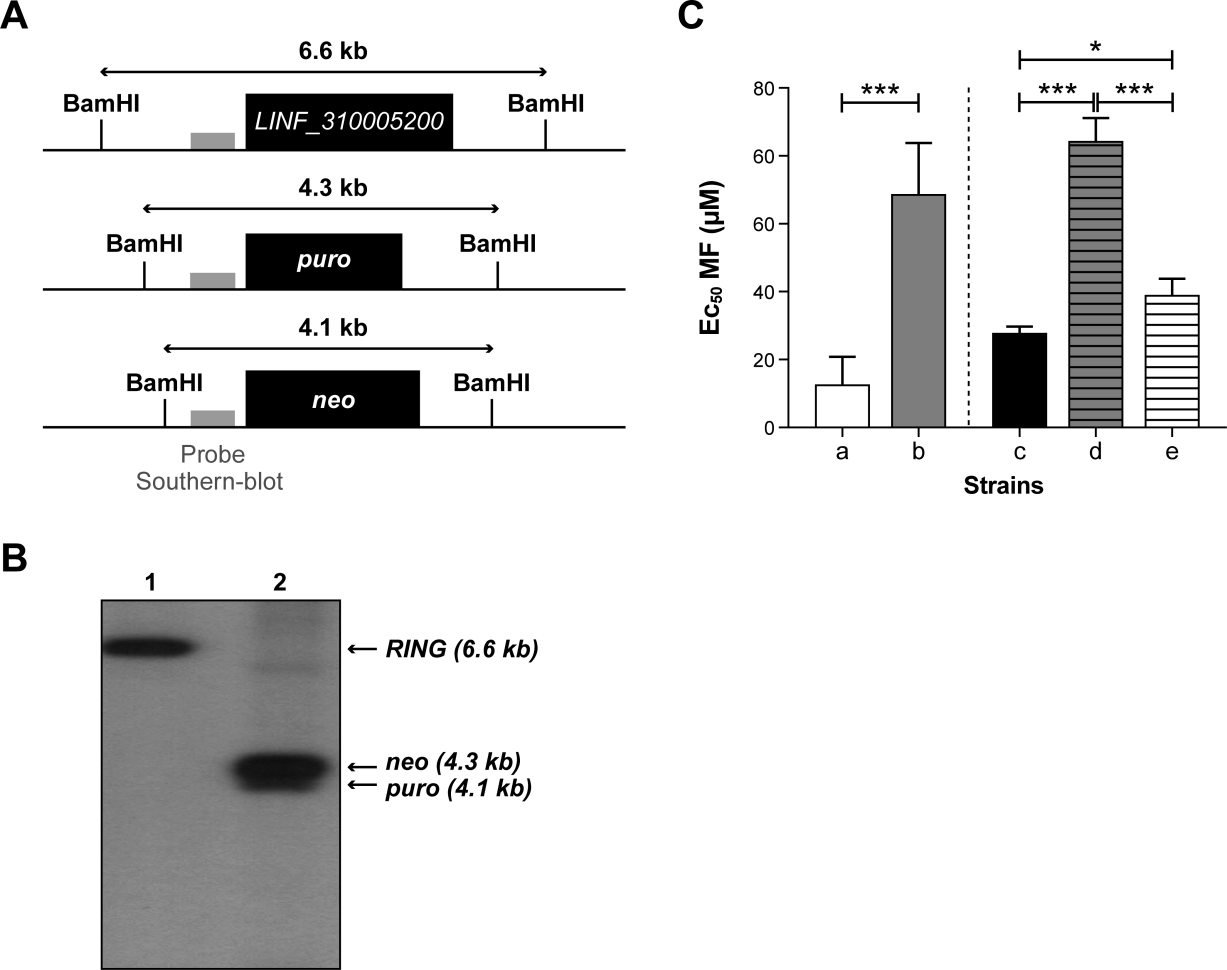
Inactivation of *LINF_310005200* leads to miltefosine resistance. (**A**) Schematic map of the *LINF_310005200* locus and of the integration of the knockout cassettes. The probe used for Southern blot hybridization is indicated by a gray box. (**B**) Hybridization of a Southern blot of *Bam*HI digested genomic DNA derived from Cas9-expressing *L. infantum* (lane 1) or a *neo/puro* null mutant for *LINF_310005200* (lane 2). (**C**) Susceptibility of *L. infantum* cells toward miltefosine. (a) Cas9-expressing *L. infantum*; (b) *L. infantum RING* null mutant; (c) Cas9-expressing *L. infantum* with a pSP72α*BLA*α vector; (d) *L. infantum RING* null mutant with a pSP72α*BLA*α vector; (e) *L. infantum RING* null mutant with an episomal *RING* add back cloned in a pSP72α*BLA*α vector. The mean and standard deviation of a minimum of three independent experiments in triplicate are shown. *P ˂ 0.05, ***P ˂ 0.001.

The MF screen led to the enrichment of three sgRNAs, one targeting the *MT* gene enriched at 10 × MF EC_50_ and one against each of the genes *LINF_310005200* and LINF_200007400 at 5 × MF EC_50_. The loss-of-function of *MT* and its role in MF resistance is firmly established (12, 23, 29, 30) hence validating our screen. The discovery of *LINF_310005200* with a RING-variant domain, and of the LINF_200007400 transmembrane protein of unknown function, potentially linked to MF is new. RING motif proteins are diverse (31, 32) and *LINF_310005200* was chosen for further functional validation. The *LINF_310005200* protein has four pairs of zinc-ion binding domains (RING finger, GO:0008270) at its N-terminus (Fig. S10A) and its RING-CH-type domain is well conserved in its *L. donovani* ortholog *LDHU3_31.0050* (Fig. S10B). Interestingly, the RING domain is known to be a part of the E3 ubiquitin ligase family (32–36) which plays a crucial role in ubiquitination, thereby influencing the stability and function of various proteins. Comparing the sequence of the RING motif of *LINF_310005200* by cross-reference entries (PROSITE entry PS51292) to the RING-CH-type domain from *LDHU3_31.0050* and 9 different MARCH family E3 ubiquitin ligases from human, bovine, frog, mouse and rat supports the predicted function of *LINF_310005200* (Fig. S10C). To assess the role of *LINF_310005200* in MF susceptibility, we integrated a *neo* and *puro* inactivation cassette (Fig. 6A) by CRISPR-Cas9 using the sgRNA LinJ.31.0040_14281-v2 (Table S3). Transfecting this sgRNA in *L. infantum* cells expressing Cas9 led to a *LINF_310005200* null mutant as confirmed by Southern blot hybridization (Fig. 6B). This cell line was fourfold more resistant to MF in comparison to the *L. infantum* Cas9 control (Fig. 6C) and adding back an episomal copy of *LINF_310005200* in this null mutant partly restored sensitivity to MF (Fig. 6C).

**Fig 7:**
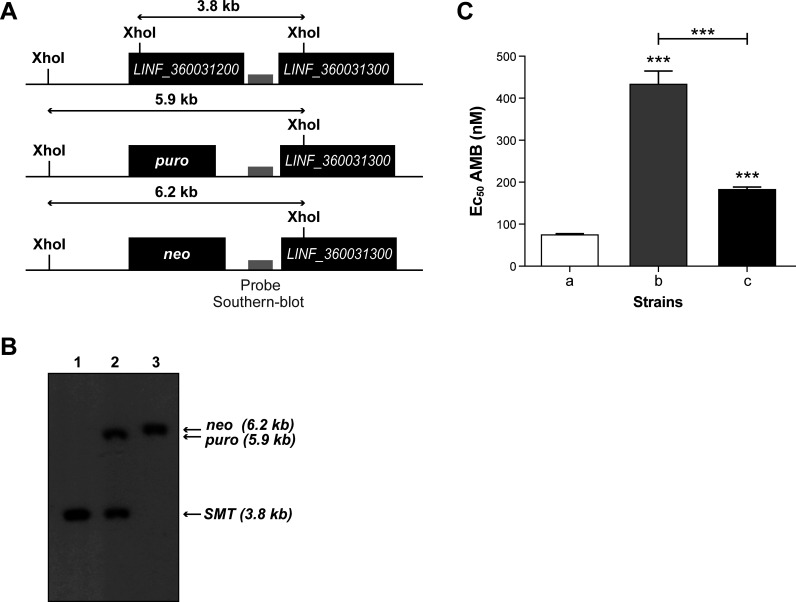
Inactivation of the *sterol 24-C methyltransferase* (*SMT*) gene leads to amphotericin B resistance. (**A**) Schematic map of the *SMT* locus and of the integration of the knockout cassettes. The probe used for Southern blot hybridization is indicated by a gray box. (**B**) Hybridization of a Southern blot of *Xho*I digested genomic DNA derived from *L. infantum* wildtype (lane 1) or from a *L. infantum SMT* single-knockout *puro* (lane 2) or *neo* null mutant (lane 3). (**C**) Susceptibility of *L. infantum* cells toward AMB. (a) Cas9-expressing *L. infantum*; (b) *L. infantum neo* null mutant; (c) *SMT neo* null mutant with an episomal *SMT* add back cloned in a psp72α*SAT*α vector. The mean and standard deviation of a minimum of three independent experiments in triplicate are shown. ***P < 0.001.

The AMB screen led to the enrichment of three sgRNAs targeting the sterol 24-C methyltransferase *SMT* genes. We transfected an sgRNA targeting the *SMT* copies (LinJ.36.2510_496-man; Table S3) along with either a *puro* or a *neo* gene inactivation cassette (Fig. 7A). One copy of the *SMT* locus was deleted with the *puro* repair cassette, while transfection with the *neo* cassette led to a null mutant as revealed by Southern blot hybridization (Fig. 7B). We measured the AMB EC_50_ of this *neo* null mutant, and it was >5-fold more resistant to AMB than the control (Fig. 7C). Adding back an *SMT* episomal copy in this mutant restored sensitivity to AMB (Fig. 7C). We also generated a null mutant of the hypothetical gene *LINF_350014000* (Fig. S11A and B) highlighted by the AMB screen (Fig. 5). The null mutant showed a twofold AMB resistance compared to the control; however, the episomal addback failed to reverse this phenotype (Fig. S11C), raising questions about the direct involvement of this hypothetical protein in AMB resistance.

## DISCUSSION

Several gain- and loss-of-function genome-wide screens have been developed for studying mode of action and resistance to drugs. An RNAi loss-of-function screen proved most useful in studying the drug mode of action in the African trypanosomes (37, 38). However, the RNAi is only functional in species from the *Leishmania Viannia* subgenus, and it is thus not active in *L. infantum* (39, 40). The CRISPR-Cas9 loss-of-function strategy has been exploited successfully in *Leishmania* (12, 13). This technology has evolved into powerful genome editing toolkits (14, 15), including barcode sequencing for high-throughput screens of pools of edited mutants (41, 42) and a cytosine base editor to introduce stop codons within open reading frames (43). All these tools have allowed the loss-of-function studies of single or multiple genes in *Leishmania*, like the characterization of 159 (mid-throughput) protein kinase mutants to establish their importance in survival, differentiation and host cell infection *in vitro* and *in vivo* in invertebrate and vertebrate hosts (42). However, strategies based on homologous recombination are not suitable for whole-genome CRISPR-Cas9 screens and instead these rely on NHEJ, the preferred pathway to repair double-strand breaks (44), produced here by Cas9. Such screens have been carried successfully in multiple organisms proficient for NHEJ, including in *Toxoplasma* parasites (16). However, *Leishmania* does not have a functional NHEJ machinery, but MMEJ is highly active (18, 19). In recent CRISPR-Cas9 work, it was shown that *Leishmania* predominantly favors single-strand annealing (SSA) over MMEJ for the repair of double-strand breaks (45), and thus, SSA may also be used when carrying out screens.

While first working with the miltefosine transporter gene *MT*, we showed that our DD vectors with six different sgRNAs could lead to MF-resistant parasites (Fig. 2C) by changing the original configuration of the *MT* gene, as suggested by the variety of PCR bands obtained when amplifying the *MT* locus (Fig. S1C and S4). This result, however, was only observed if MF selection was applied for selecting for gene editing events. Indeed, the number of cells edited is likely to be minimal since unselected cells, after one passage, were not resistant to MF (Fig. 2A). This is likely due to the suboptimal performance of the DD vector since when synthetic sgRNAs were directly transfected in Cas9-producing cells, the resulting transfectants, after one passage, were resistant to MF (Fig. 2A). Possibly a vector allowing a better expression (e.g., a cassette integrated in the genome) or processing of the cloned sgRNAs (e.g., using ribozymes) would contribute to a more effective system. We thus need a strong selective pressure with our current system, and it would be difficult to select for events that lead to subtle phenotypes or to essential genes as demonstrated with organisms having active NHEJ (16). Our pilot study with 24 sgRNAs revealed that in general few guides, often a single one, emerged after the drug selection. This is probably due to the infrequency of gene editing events and once such an event takes place, there is a clonal expansion of it. Indeed, although we cannot exclude that some sgRNAs are more effective than others, the six sgRNAs tested individually against the *MT* gene appeared similarly effective in leading to MF-resistant parasites (Fig. 2C). Attempts have been made to reconstitute an NHEJ system in *Leishmania* (46) and further necessary improvements of this strategy could eventually allow a more effective repair of double-stranded breaks when no homologous repair cassettes are provided. Nonetheless, our pilot study with the 24 sgRNAs has shown that selection with either tubercidin or MF led to the enrichment of the expected sgRNAs. We had also selected for resistance to SbIII at 5 × and 10 × the EC_50_, as our mini-library of 24 sgRNAs contained 6 guides that targeted *AQP1*, a validated marker of antimonial resistance in clinical isolates (47). We sequenced the sgRNAs in 10 clones derived from either the 5 × and 10 × selected cells and all contained the AQP1-3 sgRNA (Table S1). When we amplified *AQP1* however, we did not observe complete disappearance of the gene in these parasites but observed a partial deletion at the higher drug concentration (Fig. S12A), accompanied by an increase in resistance to SbIII (Fig. S12B). This partial deletion is probably because *AQP1* is encoded by chromosome 31, a chromosome known to be polyploid in *Leishmania*.

We next tested a genomic library of sgRNAs selected with either MF or AMB. For the MF screen, the most enriched sgRNA targeted the *MT* gene whose role in MF resistance has been firmly established by numerous studies (22, 23, 29, 45). We found another sgRNA enriched in that screen that targeted a gene coding for a RING-variant domain protein. Through functional studies we proved that a loss-of-function of this gene contributes modestly to a reduced susceptibility to MF (Fig. 6C). The sgRNA corresponding to this RING-variant domain gene was more enriched in cells selected with 5 × the EC_50_ of MF than in those selected at 10× (Fig. 4B), suggesting that this could be a first response to MF stress and once a better resistance mechanism is in place (e.g., *MT* gene deletion) there is less need to select for cells with a deletion in the RING-variant domain gene. This RING domain contains zinc fingers, a domain possibly involved in protein-protein interactions (33) and found in a variety of proteins with E3 ubiquitin ligase activity (Fig. S10C). Ubiquitin protein ligases have a relevant role in the response to drugs and have been at the basis of the development of proteolysis-targeting chimeras (PROTACs). Ubiquitination through ligases can lead either to protein degradation or to differential localization (48–51). Differential ubiquitination might affect various aspects, such as the quantity or the localization of membrane transporters. These changes, in turn, could have a significant impact on the susceptibility to MF, altering how these transporters function or where they are positioned within the cell. One such transmembrane protein could possibly correspond to the product of *LINF_200007400*, the gene targeted by the third sgRNA enriched in the MF screen. Obviously, this will require further experimental validation, the first being the confirmation that inactivation of *LINF_200007400* confers resistance to MF, but it is intriguing that the sgRNA targeting this gene showed the exact same enrichment behavior as the one targeting the RING-variant domain gene.

The screen with AMB led to three sgRNAs that were highly enriched and that were targeting the *SMT* gene, a key gene involved in ergosterol biosynthesis (52, 53), the target of AMB. Gene knockout of the *SMT* locus further confirmed that a loss-of-function of *SMT* leads to AMB resistance (54) (Fig. 7). It is not clear why, in this case, we observed more than one sgRNA targeting a specific gene. Since *SMT* is in two copies this may increase the number of sgRNAs selected or alternatively, the *SMT* sgRNAs were more properly expressed. One other sgRNA enriched in most replicates of the AMB screen targeted a gene coding for a hypothetical protein (*LINF_350014000*). A null mutant was in line with this gene contributing to AMB resistance, but we were unable to revert the phenotype. Reversal by episomal rescue is often not perfect in *Leishmania* although at the very least a partial reversion is required to ensure that a gene is truly phenotypic. Since the knockout involved Cas9-induced DSB, off-target effects cannot completely be ruled out. Also, MMEJ relies on short homology regions between DNA strands to join the broken ends, which can lead to unpredictable deletions and potentially chromosome rearrangements. This can result in a wide range of effects, impacting genetic interactions as well as gene expression. At this stage, we are unsure of the role of this hypothetical protein in the AMB susceptibility phenotype. Additional work will be required, such as reinsertion of a copy of the gene in the chromosomal locus given that the level of expression from episomes may not be optimal, as we have demonstrated previously with a gene knockout for the DNA repair protein MRE11 (55).

In summary, we have generated a genome-wide library of sgRNAs for probing the genome of *L. infantum* for genes whose loss-of-function plays a role in drug resistance. While we relied on the relatively rare event of MMEJ, we succeeded in highlighting known and new resistance genes. This library has the potential to expedite our comprehension of the mode of action and resistance mechanisms of newly investigated active scaffolds.

## MATERIALS AND METHODS

### Parasites and culture

Wild-type *Leishmania infantum* (MHOM/MA/67/ITMAP-263) parasites were maintained as promastigotes at 25°C in SDM-79 medium supplemented with 10% (vol/vol) heat-inactivated fetal bovine serum (FBS), 10 µM biopterin, and 5 µg/mL hemin at pH 7.0. Selection drugs were added at the following concentrations: 600 µg/mL hygromycin B (Wisent BioProducts), 200 µg/mL nourseothricin sulfate (GoldBio), 120 ng/µL blasticidin S HCl (Wisent BioProducts), 40 or 80 µM miltefosine (Cayman Chemical), and finally from Sigma Aldrich: 800 µg/mL paromomycin sulfate salt, 80 µg/mL G418 disulfate, 60 µg/mL puromycin dihydrochloride, 600 or 1,200 nM tubercidin, 250 or 500 µM potassium antimony(III) tartrate hydrate, and 150 or 300 nM amphotericin B.

### Construction of vectors

Primer sequences used in this study are listed in Table S1. For the expression of the *Streptococcus pyogenes* Cas9, the pSP72α*hyg*α*Cas9NLS* was generated by the amplification of Cas9 coupled with a SV40 nuclear localization sequence (NLS) and a Flag epitope tag from pLP*hygCas9* (Addgene 63555) (12). The amplified product was purified and cloned into the vector pSP72α*hyg*α (56), which contains the selectable marker hygromycin B phosphotransferase (*hyg*) gene under the control of cis-splicing elements derived from α*-tubulin* untranslated regions (UTRs).

For the expression of the sgRNA library, the DD vectors series was constructed using the low copy number plasmid p1.2*neo‐GFP* (57), which contains a rRNA polymerase I promoter (rRNAp) from *Leishmania* spp. and the neomycin phosphotransferase gene (*neo*) as the selectable marker. For the first-generation DD vector, an sgRNA expression cassette was integrated into the *Kpn*I restriction site of p1.2*neo‐GFP*. This cassette contains 20 nucleotides corresponding to the CRISPR RNA (crRNA) followed by 82 nucleotides corresponding to the trans-activating CRISPR RNA (TracrRNA) and by a Lys-tRNA gene (*LINF_030011800*) in antisense orientation used as a transcription termination signal (Fig. 1A). Flanking this sgRNA cassette are the adapters R1 and R2 (Table S1) used for sgRNA identification by Illumina sequencing (Fig. 1A). A second-generation DD vector, named DD-a, was constructed for the whole-genome screening experiment where a new sgRNA cassette synthesized as a gBlock (Integrated DNA Technologies) was integrated by *Kpn*I cloning into the DD vector. The Nextera adapter R1 and the crRNA were removed, while the TracrRNA sequence was truncated and a *Nhe*I restriction site was added for the in-frame cloning of gRNAs by Gibson assembly, by providing a free cytosine upon restriction digest that will allow reconstituting the 3′-truncated TracrRNA after assembly (Fig. S3). Cloning by Gibson assembly of the sgRNAs into the DD-a vector (see below), indeed, reconstitutes a functional sgRNA cassette almost identical to the one of the original DD (Fig. 1B).

### Library design

The set of all possible crRNAs in the genome of *L. infantum* was designed *in silico* by searching for every NGG tri-nucleotide (i.e., the protospacer adjacent motif [PAM] for Cas9) on both DNA strands and by retrieving the 20 nucleotides upstream of these PAMs. This initial set of sgRNAs was filtered to remove duplicated crRNAs and those with likely off-targets (i.e., sequences of 20 nucleotides if they had less than three mismatches with one of the crRNAs and were followed by a NAG tri-nucleotide). The resulting set crRNAs was filtered to keep only those located within coding sequences. These were then scored based on several criteria previously shown to influence gene editing efficiency (58, 59). The crRNAs were required to have ≥65% purine content, a GC content ranging from 40% to 75%, nearby microhomology sites and no TGG PAM and were used for the pilot mini-library experiments. For the whole-genome library, we selected crRNAs located in the first-half of the length of the genes, avoiding homopolymers stretches of four or more nucleotides, and favoring a cytosine at position 18 of the crRNA, i.e., at the Cas9 cleavage site (41, 58). Control crRNAs were also designed by generating a set of 500 random 20-mers not found in the *L. infantum* genome. For the whole-genome library, universal anchor sequences were added at the 5′ and 3′ ends of each sgRNA for Gibson assembly purposes, and the final set of 50,254 sgRNAs (6 sgRNAs per gene of *L. infantum* and 500 non-targeting controls) were synthesized using an on-chip *in situ* oligo-synthesizer system at CustomArray Inc (Table S3).

### Library amplification and cloning

For the mini-library into the DD vector, the sgRNA were amplified using a forward primer (crRNAML_FW) which contains a *Stu*I restriction site followed by 20 nucleotides from the crRNA (Table S1) and the sequence 5′-GTTTTAGAGCTAGAAATAGCAAGTT-3′ complementary to the TracrRNA, and the reverse primer (*Cla*I-tRNA_RV). The forward primer consisted in 24 primers directed at four genes that were mixed in an equimolar fashion. The PCR was done using the following thermal cycling parameters: 98**°**C for 2 min; 9 cycles of 98**°**C for 20 s, 72–1**°**C/cycle for 30 s, and 72°C 20 s; 20 cycles of 98**°**C for 20 s, 60–1**°**C/cycle for 30 s, and 72**°**C for 20 s; 72**°**C for 5 min. PCR products of 255 bp were run on 0.8% agarose gels, purified using a Gen Elute Gel Extraction Kit (Sigma Aldrich), restricted with *Stu*I and *Cla*I (New England Biolabs, NEB), and ligated into *Stu*I and *Cla*I-digested DD vector.

The steps for cloning the whole-genome library into the DD-a vector are summarized in Fig. S3 and S13. First, the synthesized sgRNAs pool was amplified in 10 PCRs using the Phusion polymerase (NEB) and oligonucleotides LibrF and LibrR (Table S1) with the same cycling parameters as described above. PCR products of 150 bp were run on 0.8% agarose gels and purified using the Gen Elute Gel Extraction Kit. The DD-a vector was digested with *Nhe*I (NEB), dephosphorylated with Antarctic Phosphatase (NEB), ran in 0.8% agarose gel, and finally purified with the Gen Elute Gel Extraction Kit. Twenty ligations of the amplified sgRNAs in the digested DD-a vector were performed using the Gibson assembly Master Mix (NEB, E2611) with 100 ng of vector and 20 ng of the PCR products or the same volume of water as a negative control for 30 min. The reactions were purified by isopropanol precipitation, and *E. coli* TOP10 electrocompetent bacteria were transformed using 200 ng of the library or the negative control. Approximately 8 × 10^6^ transformants were obtained, corresponding to more than 100-fold coverage of the sgRNA library. Colonies were scraped from bacterial plates, and DNA was extracted using a QIAfilter plasmid Maxi Kit (Qiagen). The abundance and evenness of sgRNA plasmids in the library were determined by next-generation sequencing (NGS). NGS-ready libraries were generated by amplifying the sgRNA cassettes by PCR using the Phusion polymerase and Illumina Nextera XT Index primers. PCR products of 438 bp were run in 0.8% agarose gels with a 1 kb Plus DNA Ladder and purified using GenElute Gel Extraction Kit to remove unincorporated primers and non-specific products, followed by quantification by a Quantus fluorometer and sequencing on an Illumina MiSeq system (2 × 150 bp paired-end reads).

### Transfections

*Leishmania* promastigotes were transfected by nucleofection using the Amaxa Cell Line Nucleofector Kit T or Amaxa P3 Primary Cell 4-D Nucleofector X Kit L (Lonza) with the U033 and E0115 programs for the Amaxa nucleofectors IIb and 4D, respectively. 5 × 10^7^ log-phase parasites were collected by centrifugation and resuspended in 100 µL of the kit solutions with the appropriate nucleic acid (plasmids, sgRNA, and/or DNA repair templates). After transfection, parasites were allowed to recover for 24 h before the addition of the appropriate selection marker drugs. These pre-selected parasites were then sub-cultured 24 h later in the presence of marker drug selection in fresh medium. Negative controls corresponding to transfections with an empty vector or without sgRNA were included.

### Libraries screening

Twenty-five transfections with 5 µg of DD-a sgRNA library were done in *L. infantum* expressing Cas9. After 24 h, paromomycin and hygromycin were added for the selection of the DD-a plasmids and for maintenance of the Cas9 episome, respectively. The 25 transfections were pooled 24 h later, leading to a master culture that was passaged in fresh medium at a density of 1.8 × 10^7^ parasites/mL in the presence of G418 disulfate and hygromycin. Once the cells have reached OD 0.5–0.6 (approximately 5 days), the cultures are divided at the same density, supplemented in fresh culture medium and selected with either 5× or 10× the EC_50_ of MF. For AMB screening, the cells are first placed in the presence of 5× the EC_50_ of AMB (day 5) then, once the OD 0.5–0.6 is reached, a new passage is carried out in the presence of 10× the EC_50_, in biological triplicate (day 10). Unselected transfected cultures were extracted at each passage as a control (baseline, day 5 and day 10). Sequencing the gRNAs extracted from baseline transfectants validated the evenness in coverage and abundance for the sgRNA library. Parasites were recovered from aliquots of drug-selected or control cultures by centrifugation at 3,000 rpm (or 850 × *g*) for 5 min, and their DD-a plasmids extracted using the Wizard Plus SV Minipreps DNA purification System (Promega).

### sgRNA sequencing

The sgRNA sequences in the DD-a plasmids were amplified with the Phusion polymerase and GC-rich 5 × Phusion buffer as described above, using the Illumina DNA/RNA UD Indexes. We used the following thermal cycling parameters: 98**°**C for 2 min; 9 cycles of 98**°**C for 20 s, 72–1**°**C/cycle for 30 s, 72**°**C for 20 s; 10 cycles of 98**°**C for 20 s, 62–1**°**C/cycle for 30 s, 72**°**C for 20 s; 72**°**C for 5 min. The amplification was done with two independent PCRs for each replicate, each with a different DNA barcode. PCR products were purified by gel extraction quantified with a Quantus fluorometer and sequenced on an Illumina NovaSeq6000 platform.

### Bioinformatics

FASTQ files were demultiplexed into individual libraries using the DNA barcodes. Reads from PCR replicates of the same sample were merged into a single FASTQ file for downstream analysis. Reads obtained from the day 0 sample were used as the baseline for the library. The sgRNA sequences were extracted from the reads and classified as perfect or imperfect using a custom Python script adapted from Joung et al. (60). Imperfect sgRNAs, i.e., those having mismatches in their crRNA or TracrRNA were excluded from the analysis. The sgRNA counts in each sample were determined using a custom Python script. The identification of positively selected sgRNAs and associated statistics was computed using the RRA and MLE algorithms from the Model-based Analysis of Genome-wide CRISPR/Cas9 Knockout (MAGeCK) software (61).

### Functional validation of candidate genes pinpointed by the CRISPR-Cas screens

Single or double knock-out lines were generated by CRISPR-Cas9 using either synthesized crRNAs and TracrRNA (Integrated DNA Technologies) or using the DD vector. For synthetic guides, 5 µL of synthesized crRNA and TracrRNA (both at 100 µM) was mixed, hybridized for 5 min at 95–100°C, and incubated at room temperature for 15 min, leading to sgRNA. For the DD vector, individual plasmids carrying crRNAs targeting the candidate genes were constructed using the primers listed in Table S1. Homology repair templates (HRT) were amplified using oligonucleotides specific to the *puromycin acetyltransferase* or *neomycin phosphotransferase* genes but whose 5′ end matched the 30 nucleotides immediately flanking the 5′ or 3′ ends of the gene of interest in the genome of *L. infantum* (Table S1). For transfection, 8 µg of one or two repair templates was transfected simultaneously with 8 µL of synthetic sgRNA or 5 µg of DD vector using an Amaxa-Nucleofector. Selection was done with puromycin or/and G418, and allelic substitutions were confirmed by PCR amplification of the target gene followed by Southern blots. For Southern blots, genomic DNA was extracted from *Leishmania* cells using the DNAzol reagent following manufacturer’s instructions (Invitrogen) and digested with specific restriction enzymes (*BamH*I, *Xho*I, or *Hind*III). Digested DNA fragments were separated by electrophoresis on agarose gels, transferred to hybond-N nylon membranes (Amersham) by capillarity overnight, and hybridized at 65°C for at least 12 h with appropriate DNA probes (Table S1) amplified by PCR and labeled using [α-^32^P]dCTP, random oligonucleotides, and the Klenow enzyme (NEB).

The coding sequences of candidate genes were amplified using primers listed in Table S1, gel-purified, and cloned into the plasmid pSP72α*SAT*α or pSP72α*BLA*α (SAT, nourseothricin *N*-acetyl transferase; BLA, blasticidin S deaminase) (56). Fifty micrograms of each plasmid was used to transfect *L. infantum* parasites by electroporation, as described previously (56). Empty vectors were used as controls. The EC_50s_ of the various knockout and add-back lines generated were calculated by exposure to a range of drug concentrations in 24-well plate or T75 flasks and 5 × 10^5^ or 5 × 10^6^ mid-log phase parasites inoculum, respectively. Plates were incubated in an automated Biospa incubator, and parasite growth was monitored at 600 nm in a BioTek Cytation5 (Agilent Technologies) for 72 h. For flasks, growth was monitored at 600 nm at the 72 h end-point. The EC_50_ values were calculated using GraphPad Prism 5 software. Statistical significance between conditions was performed using the unpaired *t*-test.

## SUPPLEMENTAL MATERIAL

Supplemental Figures mbio.00477-24-s0001.docx Figures S1 to S13.

Table S1 mbio.00477-24-s0002.xlsx PCR primers and crRNA sequences for mini-library.

Table S2 mbio.00477-24-s0003.xlsx crRNAs detected upon selection of the mini-libraries with drugs.

Table S3 mbio.00477-24-s0004.xlsx crRNA sequences for the whole genome gRNA library.

Table S4 mbio.00477-24-s0005.xlsx gRNA counts upon selection of the whole genome library with drugs.

ASM does not own the copyrights to Supplemental Material that may be linked to, or accessed through, an article. The authors have granted ASM a non-exclusive, world-wide license to publish the Supplemental Material files. Please contact the corresponding author directly for reuse.
