# Supplementary material for: CRISPR-Cas9 high-throughput screening to study drug resistance in Leishmania infantum
Source: mBio. 2024 Jun 12;15(7):e00477-24. doi: 10.1128/mbio.00477-24 (PMC11253630; doi:10.1128/mbio.00477-24)
Supplement: Supplemental Figures — Figures S1 to S13. [file mbio.00477-24-s0001.docx]

**
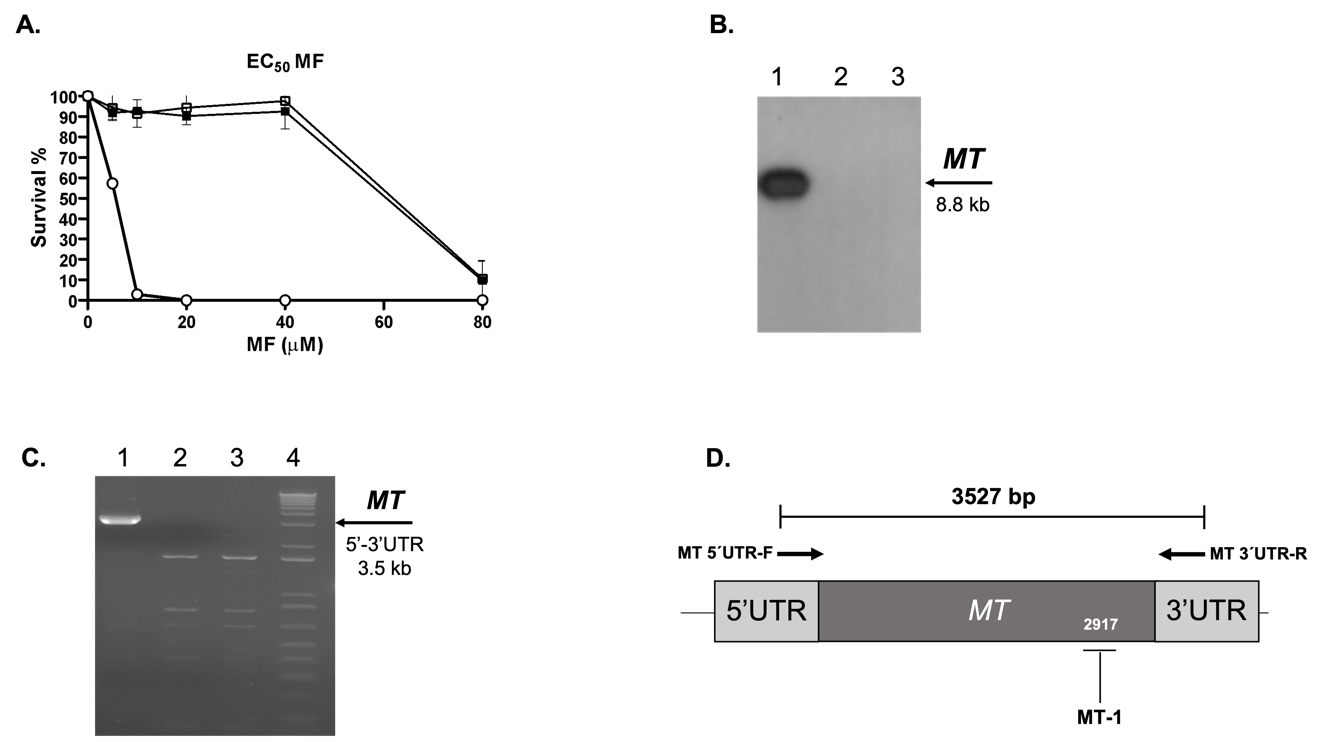
**

**Figure S1. Targeting the *miltefosine transporter* gene *MT* using the DD vector for CRISPR-Cas9 mediated gene deletion**. **A.** Miltefosine susceptibility of Cas9-expressing *L. infantum* (⭘); of Cas9-expressing *L. infantum* transfected with DD-MT-1 selected with G418 and 5× MF EC_50_ (🞎) or with G418 and 10× MF EC_50_ (◼). All growth curves were done in duplicates with four technical replicates. **B.** Southern blot of genomic DNA digested with *Xho*I, from Cas9-expressing *L. infantum* (lane 1); of Cas9-expressing *L. infantum* transfected with DD-MT-1 and selected with 5× MF EC_50_ (lane 2) or 10× MF EC_50_ (lane 3) and hybridized with a probe covering the *MT* gene. **C.** PCR amplification *MT* using primers annealing to the 5’ and 3’ untranslated regions (UTR) of the gene. Cas9-expressing *L. infantum* (1); Cas9-expressing *L. infantum* transfected with DD-MT-1 and selected with G418 and 5× MF EC_50_ (2) or with G418 and 10× MF EC_50_ (3); 1kb+ DNA ladder (4). **D.** Schematic representation of the *MT* locus with the position of the MT-1 sgRNA indicated. The position of the primers (see Table S1) used for PCR amplification of *MT* is shown by arrows.


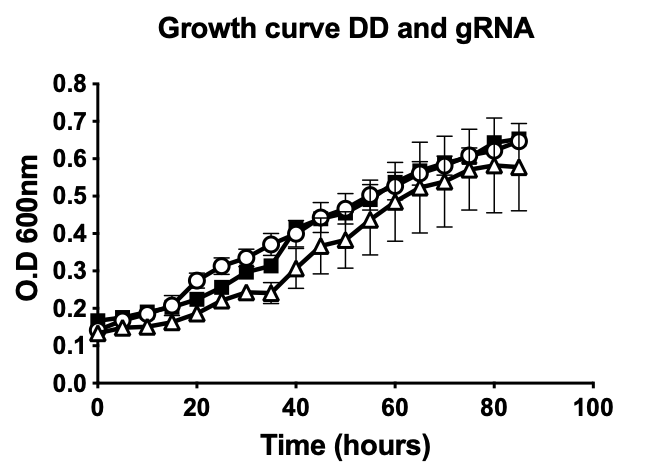


**Figure S2.** **The DD vector does not affect the growth of *L. infantum***. Growth of Cas9-expressing *L. infantum* (⭘); of Cas9-expressing *L. infantum* transfected with DD-MT-1 (◼); of Cas9-expressing *L. infantum* transfected with a synthetic MT-1 crRNA (and annealed tracrRNA) (△). Growth was monitored at 600nm every 5 hours in a Cytation5 multimode reader.


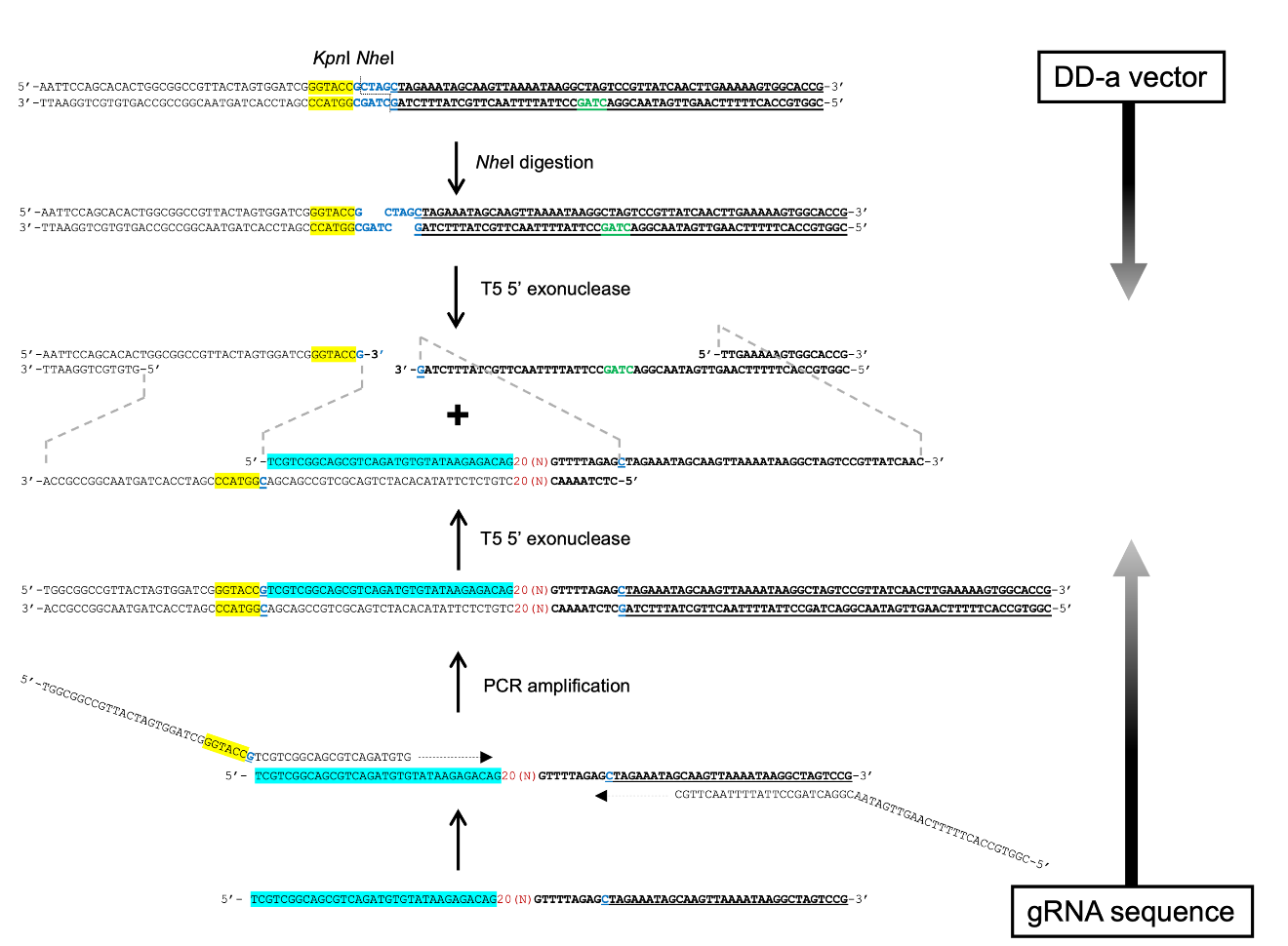


**Figure S3. Cloning of the sgRNAs library by Gibson assembly.** The DD-a vector has a *Nhe*I restriction site for cloning gRNAs just upstream of the truncated TracrRNA sequence. The pool of synthetic gRNAs is amplified using universal primers LibrF and LibrR (see Table S1). The digested DD-a and the PCR products are digested by T5-exonuclease, which produces complementary single strand DNA for the annealing of DD-a and the PCR products. The ‘C’ (in blue all along the figure) at the last position of the NheI restriction site in DD-a allows to reconstruct an in-frame and full length TracrRNA upon cloning of the gRNAs by Gibson assembly.


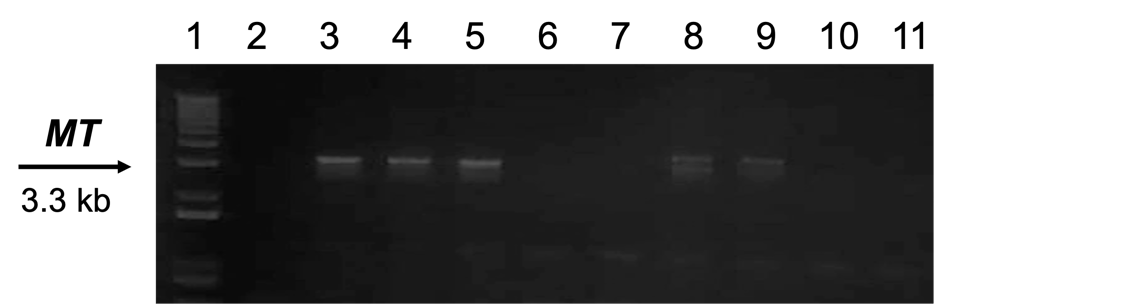


**Figure S4. PCR amplification of the MT open reading frame.** 1, 1kb+ DNA ladder; 2, water; 3, L. infantum; 4, Cas9-expressing L. infantum; Cas9-expressing L. infantum transfected with DD-a-MT1 and selected with G418 (5); Cas9-expressing L. infantum transfected with DD-a-MT-1 (6), DD-a-MT-2 (7), DD-a-MT-3 (8), DD-a-MT-4 (9), DD-a-MT-5 (10) or DD-a-MT-6 (11), all selected with G418 and 10× the MF EC50.

**
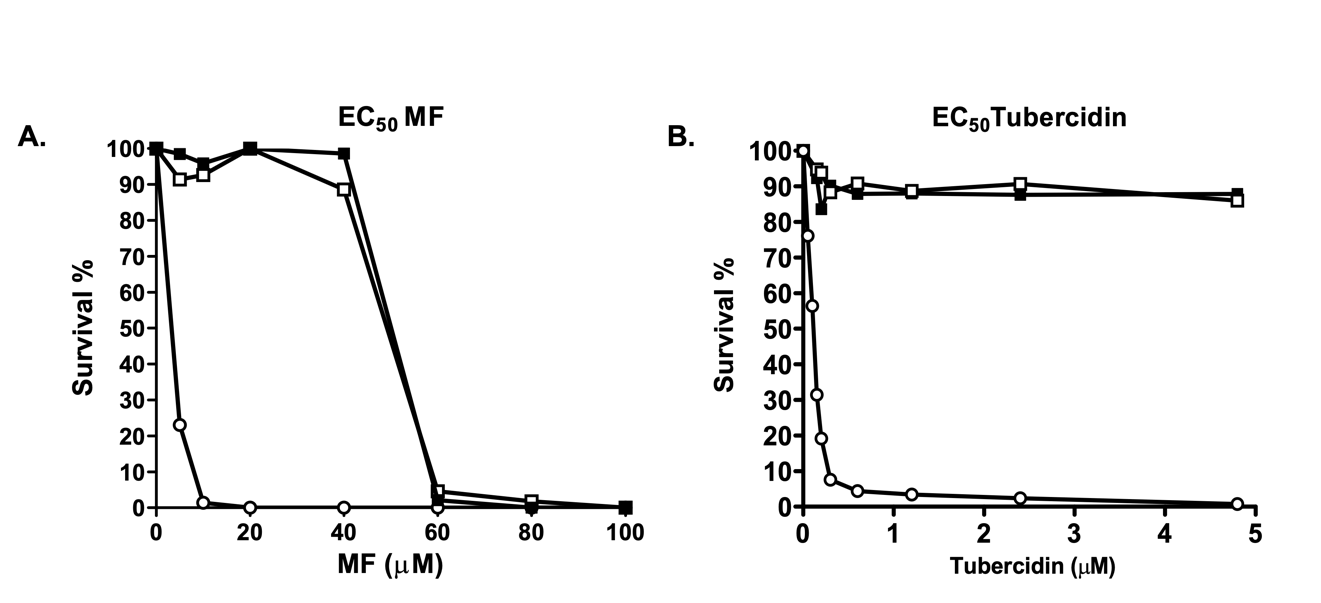
**

**Figure S5**. **Susceptibility of parasite populations subjected to a small scale dominant negative CRISPR-Cas9 screen.** **A.** Miltefosine susceptibility of Cas9- expressing *L. infantum* (⭘); of Cas9-expressing *L. infantum* transfected with the 24 sgRNAs library selected with 5× (🞎) or 10× the MF EC_50_ (◼). **B.** Tubercidin susceptibility of Cas9-expressing *L. infantum* (⭘); of Cas9-expressing *L. infantum* transfected with the 24 sgRNAs library selected with 5× (🞎) or 10× the tubercidin EC_50_ (◼).


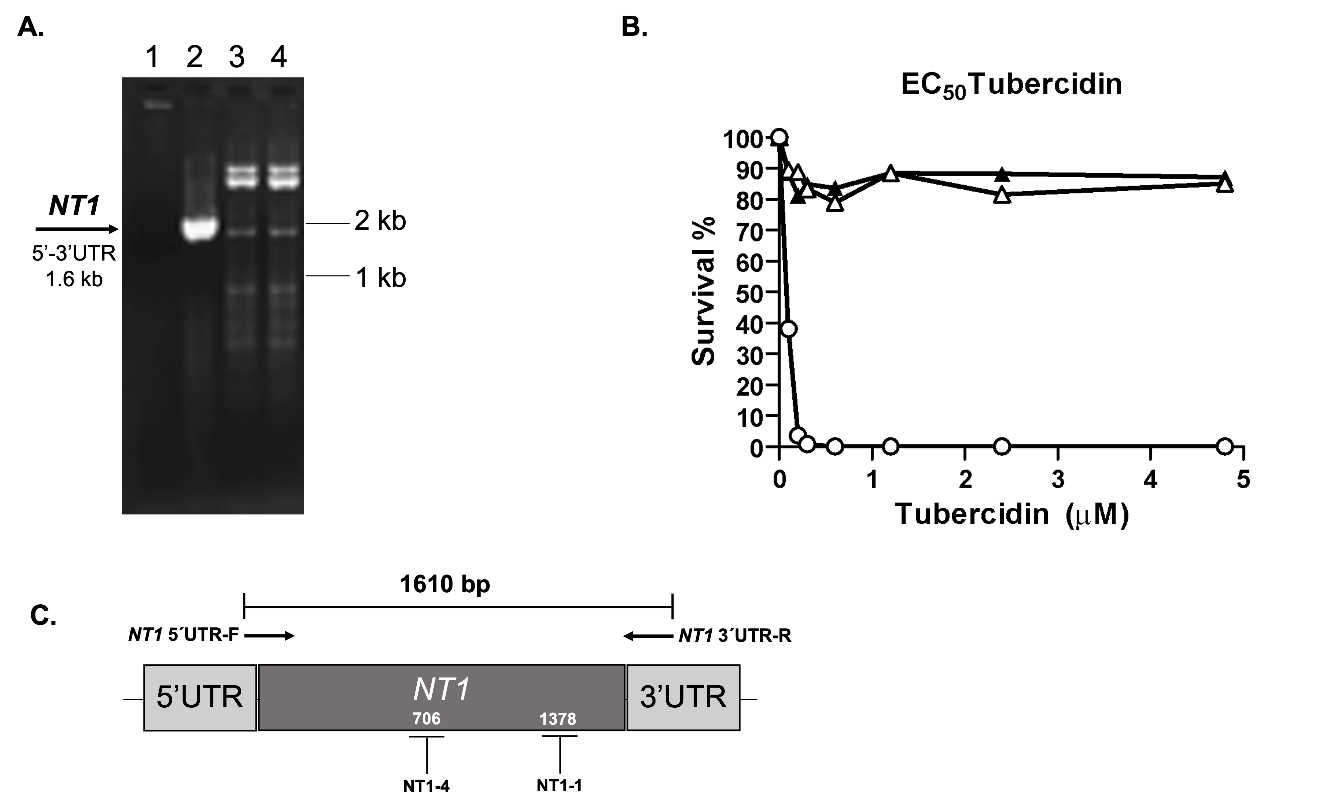


**Figure S6. CRISPR-Cas9 screening with tubercidin.** **A.** The genomic DNA of *L. infantum* (expressing Cas9) transfected with DD-a vectors expressing sgRNA targeting the *NT1* gene and selected with tubercidin was amplified by PCR for *NT1*. Water (1); Cas9-expressing *L. infantum* (2); Cas9-expressing *L. infantum* transfected with DD-a-NT1-1 (3) or DD-a-NT1-4 (4) selected with G418 and 10× the tubercidin EC_50_. **B.** Tubercidin susceptibility of Cas9-expressing *L. infantum* (⭘); of Cas9-expressing *L. infantum* transfected with DD-a-NT1-1 (△) and DD-a-NT1-4 (▲) selected with G418 and 10× tubercidin EC_50_ after transfection. All growth curves were done once with four technical replicates and the standard deviation (SD) was included. **C.** Schematic representation of the *NT1* locus with the position of the NT1-1 and NT1-4 sgRNAs (tested in panel A) indicated. PCR primers (Table S1) used for the amplification of *NT1* are shown by arrows.


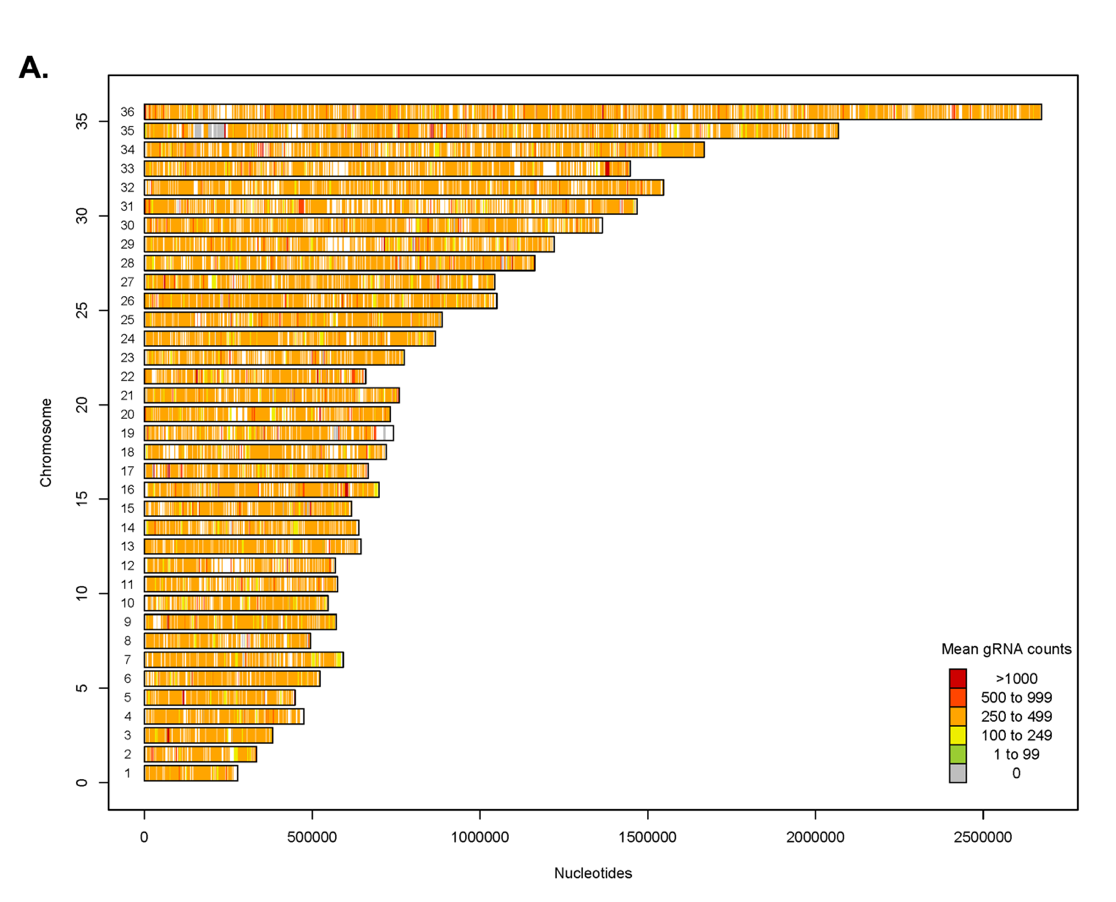


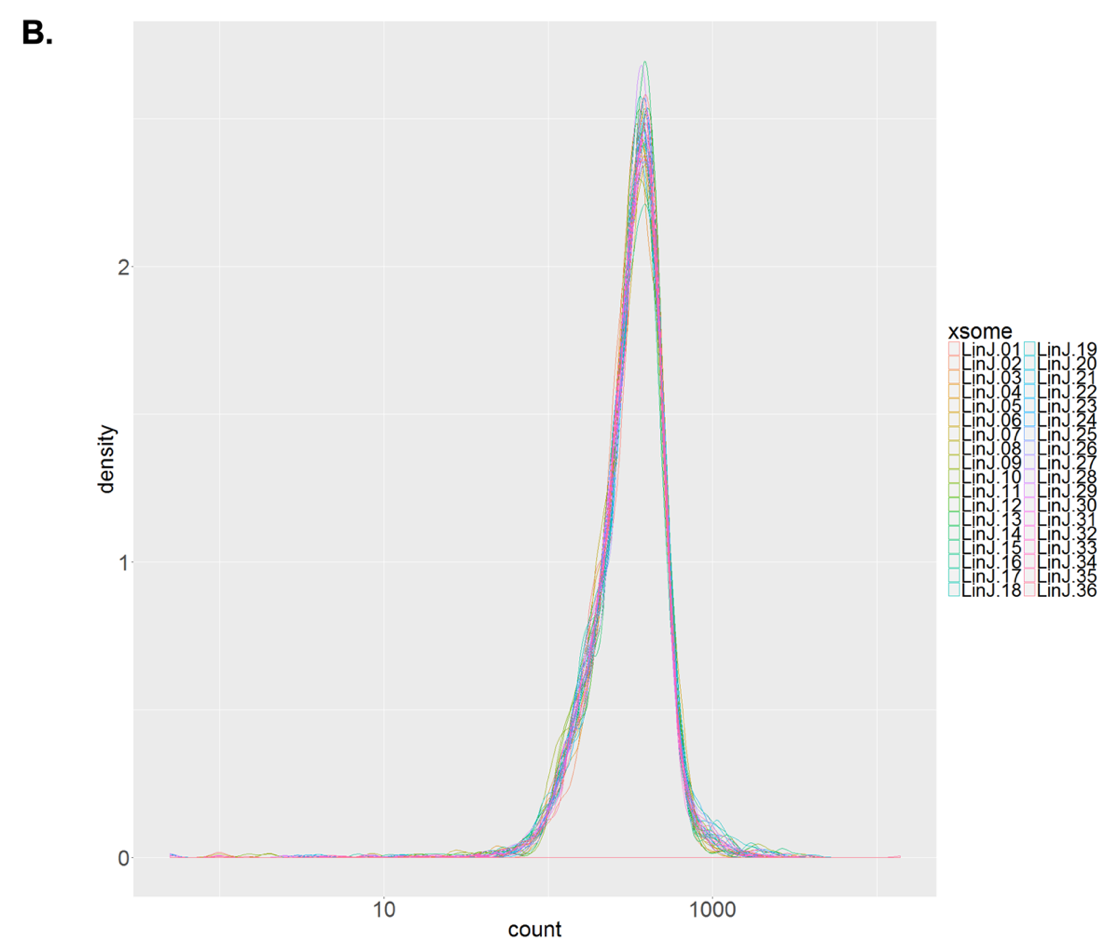


**Figure S7.** **Illustration of the balanced distribution and abundance of the whole-genome library in *Leishmania infantum*, without drug selection.** **A.** The illustration displays the positioning and abundance of sgRNAs retrieved from DD-a vectors through next-generation sequencing on a map depicting the mean sgRNA counts across the 36 chromosomes of *L. infantum* after their transfection into the parasites. Colored bars indicate mean sgRNA count according to the color code provided below, per positions on each chromosome. Over 95% of the sgRNAs from the library were detected among transfectants. **B.** Evenness of the baseline library represented by read counts per sgRNA. The uniformity of the library is represented by the number of reads per sgRNA. The skew ratio between the top 10% most abundant sgRNAs to the 10% less abundant sgRNAs was around 3, indicating a narrow distribution of read counts between sgRNAs and library evenness among these baseline transfectants.


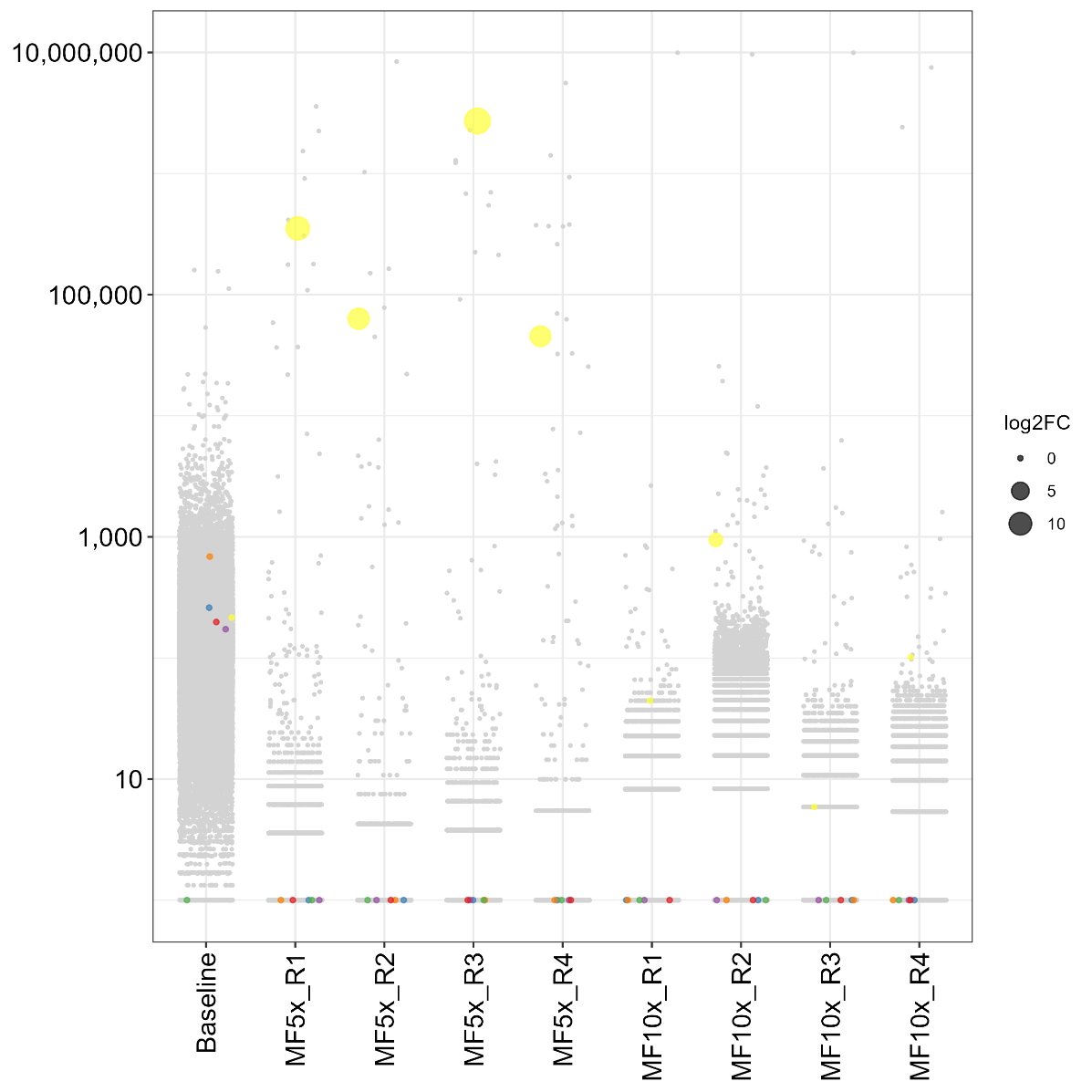


**Figure S8.** **Enrichment of a sgRNA targeting** **LINF_200007400** **in the** **CRISPR-Cas9 whole genome screen selected for miltefosine resistance.** The abundance of sgRNAs identified by sequencing the DD-a vectors recovered from transfected parasites at baseline or after selection with 5× and 10× the EC_50_ of MF are shown. The screen was performed in four replicates (R1-R4). Each dot represents a sgRNA whose abundance in read count (normalized for library size to 10M total reads) is indicated by the y-axis. Colored dots indicate sgRNAs targeting the LINF_200007400 gene, one color for each of the 6 different sgRNAs targeting the genes. The coloring scheme is independent from the one used in Figures 4 and 5. The log2 fold-change (log2FC) in abundance for these sgRNAs compared to the baseline is shown by dot size. Grey dots correspond to the bulk of sgRNAs.


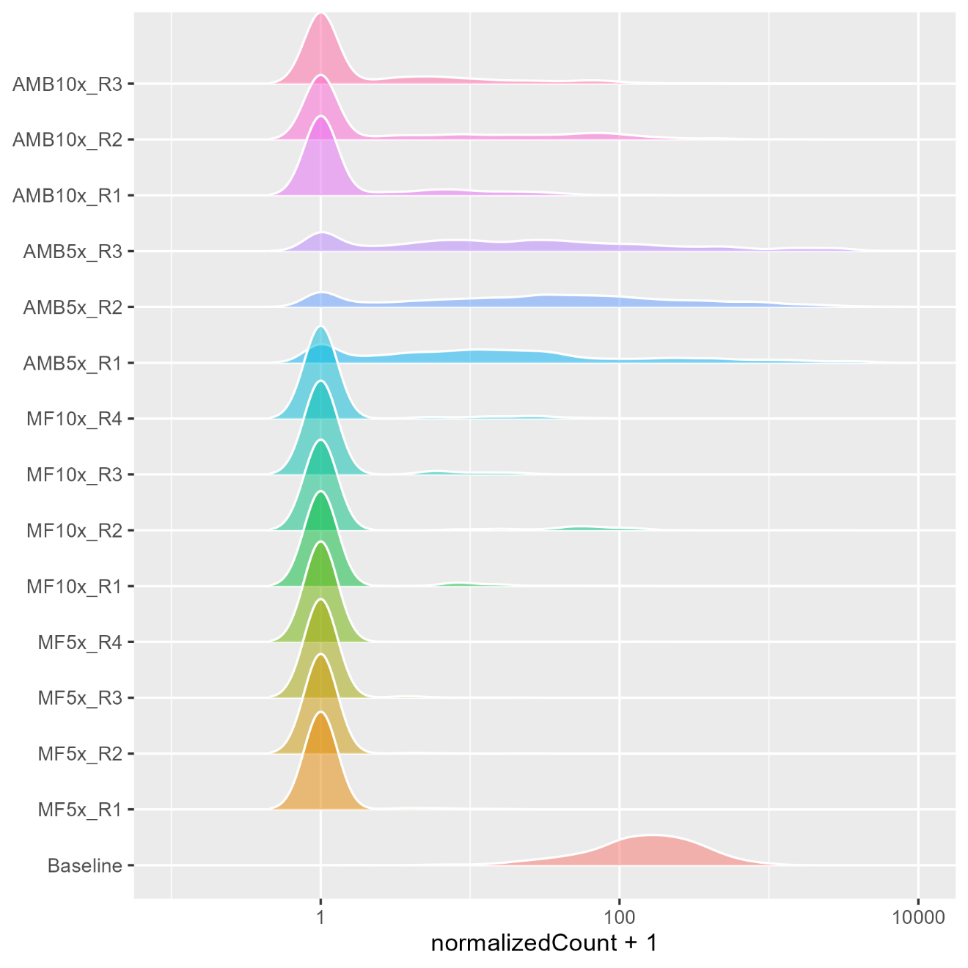


**Figure S9.** The distribution of counts (normalized for library size to 10M reads) for the 500 non-targeting control gRNAs is shown for baseline and for samples selected with MF or AMB at 5× or 10× their EC50.

**
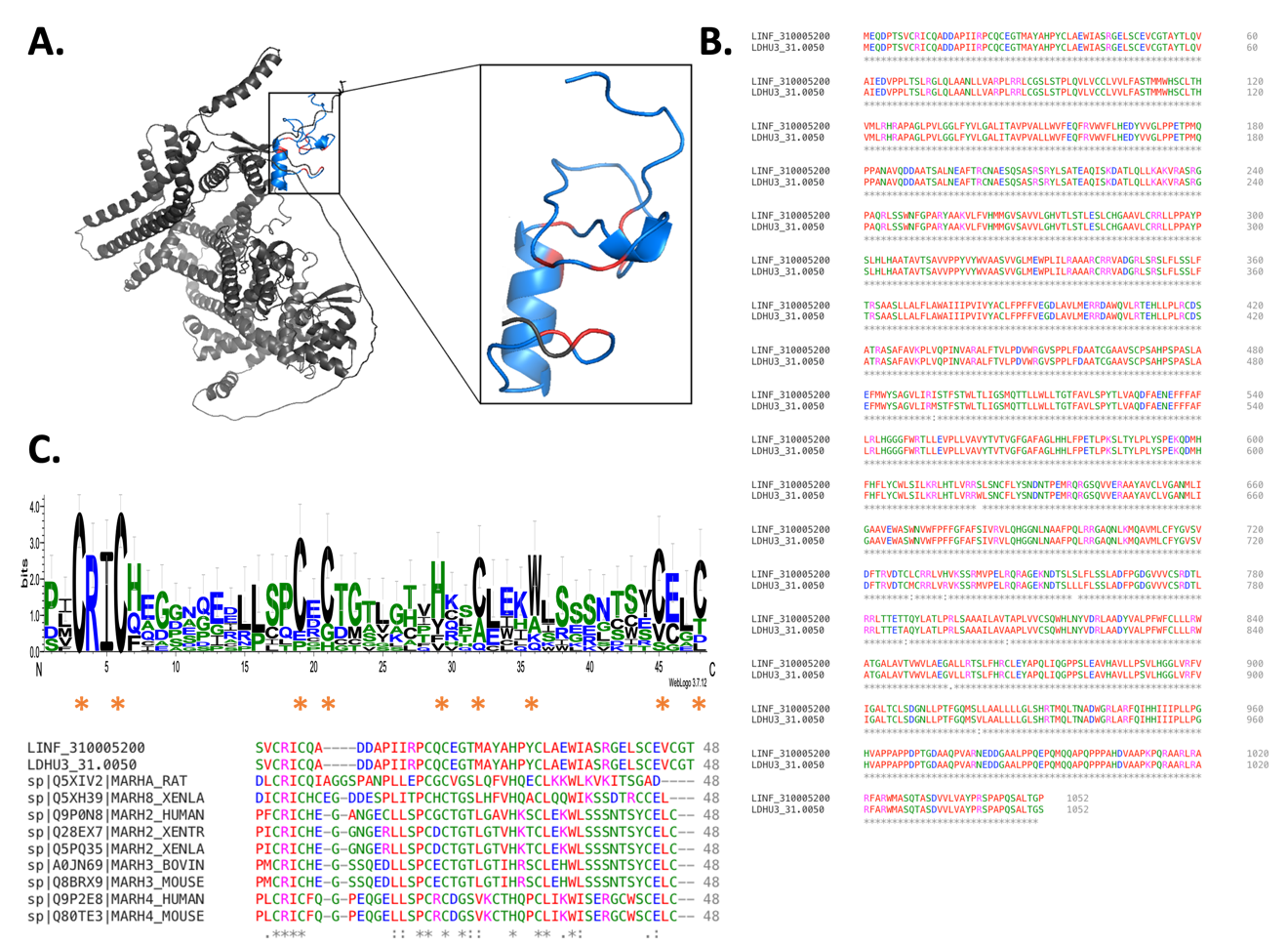
**

**Figure S10.** ***LINF_310005200* codes for a RING-variant domain protein and contains zinc fingers, a domain possibly involved in protein-protein interactions.** **A.** The *LINF_310005200* predicted protein structure was obtained using AlphaFold and visualized in PyMOL (<https://pymol.org/2/>). The RING-variant domain (Pfam PF12906) is highlighted in blue in the structure and corresponds to amino acids 1-52. The amino acids colored in red correspond to the RING-CH-type consensus sequence C-X_2_-C-X_[9-39]_-C-X_[1-3]_-H-X_[2-3]_-C/H-X_2_-C-X_[4-48]_-C-X_2_-C (C and H, conserved cysteine and histidine residues involved in zinc coordination, respectively; X, other amino acid residues). **B.** Sequence alignment of RING-CH-type domain-containing protein *LDHU3_31.0050* and *LINF_310005200* by Clustal Omega (61) **C.** Sequence alignment of the RING-CH-type motif of *LINF_310005200* and its *Leishmania donovani* ortholog *LDHU3_31.0050* with 9 Zinc finger RING-CH-type E3 ubiquitin ligases MARCH representatives from human, rat, bovine, mouse and frog. The sequence logo above the alignment shows that the most conserved amino acids of the RING motif (indicated with orange stars) are conserved in the *Leishmania* sequences.


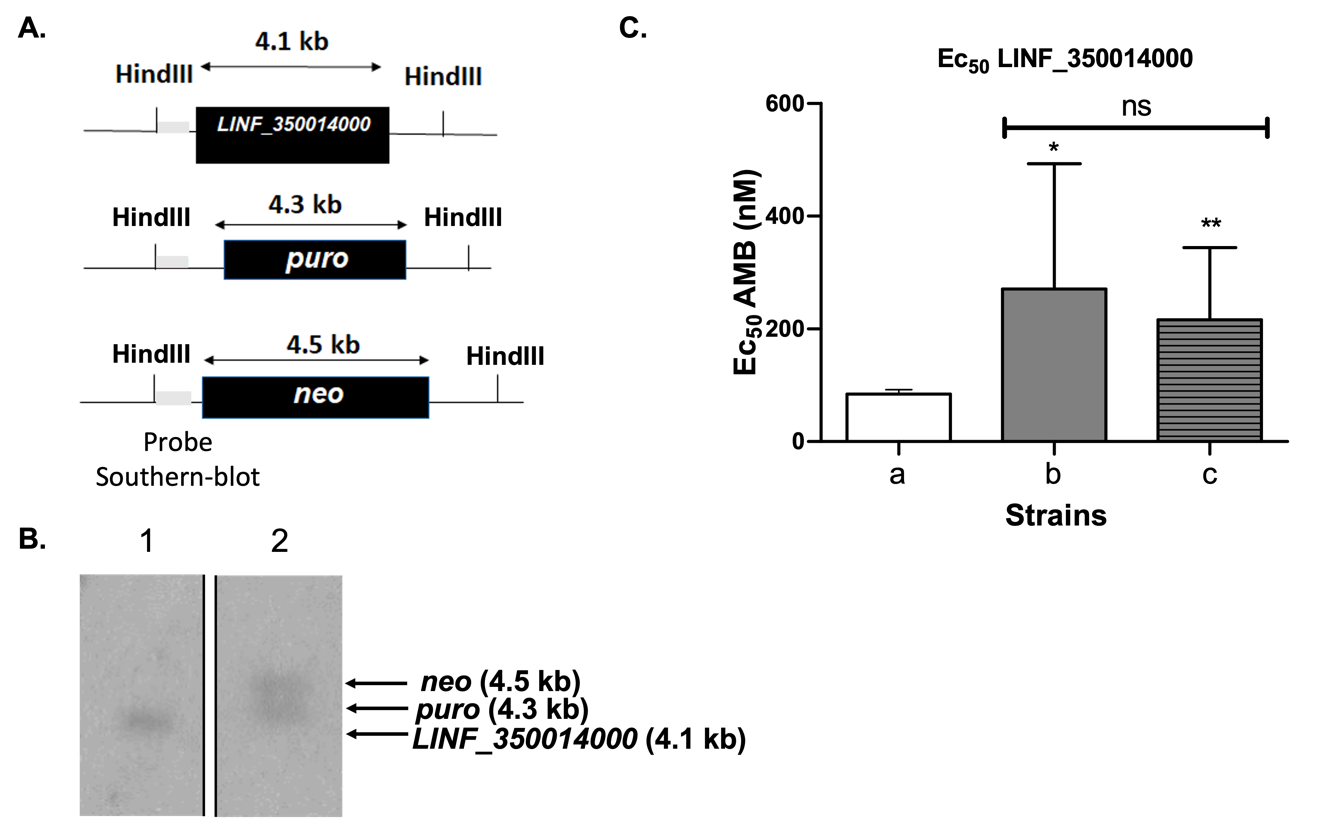


**Figure S11. Inactivation of the gene coding for the hypothetical protein (*LINF_350014000*) and its role in amphotericin B resistance.** **A.** Schematic map of the *LINF_350014000* locus and of the integration of the knockout cassettes. The probe used for Southern blot hybridization is indicated by a cyan box. **B.** Southern-blot of *Hind*III-digested genomic DNA derived from Cas9-expressing *L. infantum* (lane 1) and of *L. infantum* *neo/puro* null mutant for *LINF_350014000* (lane 2). The white band between the 2 lanes indicates where the film was cut to avoid cluttering the figure with additional irrelevant lanes. **C.** Susceptibility of *L. infantum* cells towards AMB. a, Cas9-expressing *L. infantum*; b, *L. infantum neo/puro* null mutant for *LINF_350014000; c, L. infantum neo/puro* null mutant for *LINF_350014000* with an episomal*LINF_350014000* add back cloned in a psp72*αSATα* vector.


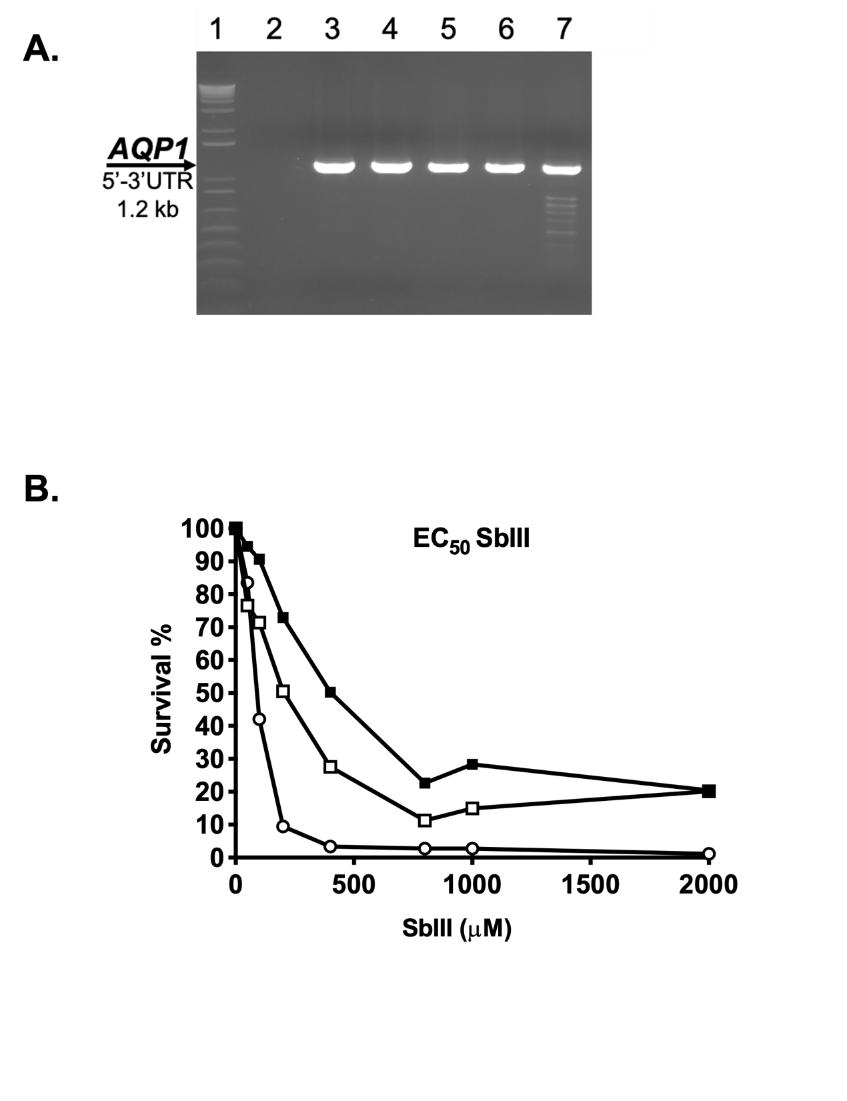


**Figure S12. Pilot CRISPR-Cas9 screen selected with trivalent antimonials.** A mini library of 24 sgRNAs cloned into the DD vector was transfected in Cas9 expressing *L. infantum* and selected with drugs. **A.** The genomic DNAs of growing parasites were used as templates for the amplification of *AQP1* using primers annealing to its untranslated regions. 1, 1kb+ DNA ladder; 2, water; 3, Cas9-expressing *L. infantum*; the Cas9-expressing *L. infantum* transfected with the DD library selected with 10× MF EC_50_ (4), with 10× tubercidin EC_50_ (5), with 5× SbIII EC_50_ (6) or with 10× SbIII EC_50_ (7). **B.** SbIII susceptibility of Cas9 expressing *L. infantum* (⭘); of Cas9 expressing *L. infantum* transfected with the 24 sgRNAs library and selected with 5× SbIII EC_50_ (🞎) or 10× SbIII EC_50_ (◼) after transfection.

**
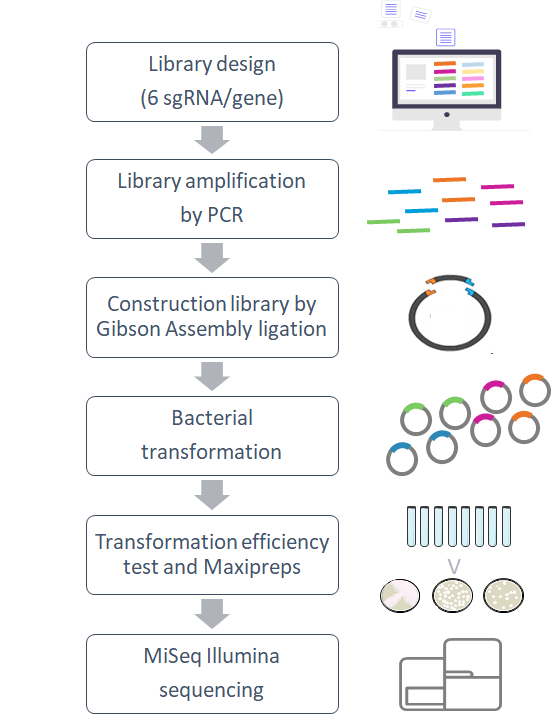
**

**Figure S13. General workflow for genome-scale CRISPR library construction.**
